# The genome of the soybean cyst nematode (*Heterodera glycines*) reveals complex patterns of duplications involved in the evolution of parasitism genes

**DOI:** 10.1186/s12864-019-5485-8

**Published:** 2019-02-07

**Authors:** Rick Masonbrink, Tom R. Maier, Usha Muppirala, Arun S. Seetharam, Etienne Lord, Parijat S. Juvale, Jeremy Schmutz, Nathan T. Johnson, Dmitry Korkin, Melissa G. Mitchum, Benjamin Mimee, Sebastian Eves-van den Akker, Matthew Hudson, Andrew J. Severin, Thomas J. Baum

**Affiliations:** 1 Department of Plant Pathology, Iowa State University, Ames, IA USA; 2 Genome Informatics Facility, Iowa State University, Ames, IA USA; 3 Agriculture and Agri-Food Canada, Saint-Jean-sur-Richelieu, QC Canada; 4 Department of Energy, Joint Genome Institute, Walnut Creek, CA USA; 5 HudsonAlpha Institute for Biotechnology, Huntsville, AL USA; 6 Bioinformatics and Computational Biology Program, Worcester Polytechnic Institute, Worcester, MA USA; 7 Department of Computer Science, Worcester Polytechnic Institute, Worcester, MA USA; 8 Division of Plant Sciences, University of Missouri, Columbia, MO USA; 9 Department of Plant Sciences, University of Cambridge, Cambridge, UK; 10 Department of Crop Sciences University of Illinois, Urbana, IL USA

**Keywords:** *Heterodera glycines*, SCN, Soybean cyst nematode, Genome, Tandem duplication, Effector, Evolution

## Abstract

**Background:**

*Heterodera glycines*, commonly referred to as the soybean cyst nematode (SCN), is an obligatory and sedentary plant parasite that causes over a billion-dollar yield loss to soybean production annually. Although there are genetic determinants that render soybean plants resistant to certain nematode genotypes, resistant soybean cultivars are increasingly ineffective because their multi-year usage has selected for virulent *H. glycines* populations. The parasitic success of *H. glycines* relies on the comprehensive re-engineering of an infection site into a syncytium, as well as the long-term suppression of host defense to ensure syncytial viability. At the forefront of these complex molecular interactions are effectors, the proteins secreted by *H. glycines* into host root tissues. The mechanisms of effector acquisition, diversification, and selection need to be understood before effective control strategies can be developed, but the lack of an annotated genome has been a major roadblock.

**Results:**

Here, we use PacBio long-read technology to assemble a *H. glycines* genome of 738 contigs into 123 Mb with annotations for 29,769 genes. The genome contains significant numbers of repeats (34%), tandem duplicates (18.7 Mb), and horizontal gene transfer events (151 genes). A large number of putative effectors (431 genes) were identified in the genome, many of which were found in transposons.

**Conclusions:**

This advance provides a glimpse into the host and parasite interplay by revealing a diversity of mechanisms that give rise to virulence genes in the soybean cyst nematode, including: tandem duplications containing over a fifth of the total gene count, virulence genes hitchhiking in transposons, and 107 horizontal gene transfers not reported in other plant parasitic nematodes thus far. Through extensive characterization of the *H. glycines* genome, we provide new insights into *H. glycines* biology and shed light onto the mystery underlying complex host-parasite interactions. This genome sequence is an important prerequisite to enable work towards generating new resistance or control measures against *H. glycines*.

**Electronic supplementary material:**

The online version of this article (10.1186/s12864-019-5485-8) contains supplementary material, which is available to authorized users.

## Background

The soybean cyst nematode (SCN) *Heterodera glycines* is considered the most damaging pest of soybean and poses a serious threat to a sustainable soybean industry [1]. *H. glycines* management relies on crop rotations, nematode resistant crop varieties, and a panel of biological and chemical seed treatments. However, cyst nematodes withstand adverse conditions and remain dormant for extended periods of time, and therefore, are difficult to control. Furthermore, the overuse of resistant soybean varieties has stimulated the proliferation of virulent nematode populations that can infect these varieties [2]. Hence, there continues to be a strong need to identify, develop, and implement novel sources of nematode resistance and management strategies.

*H. glycines* nematodes are obligate endoparasites of soybean roots. Once they emerge from eggs in the soil, they find nearby soybean roots and penetrate the plant tissue where they migrate in search for a suitable feeding location near the vascular cylinder. The now sedentary *H. glycines* convert adjacent root cells into specialized, fused cells that form the feeding site, termed syncytium [3]. The parasitic success of *H. glycines* depends on the formation and long-term maintenance of the syncytium, which serves as the sole source of nutrition for the remainder of its life cycle. Host finding, root penetration, syncytium induction, and the long-term successful suppression of host defenses are all examples of adaptation to a parasitic lifestyle. At the base of these adaptations lies a group of nematode proteins that are secreted into plant cells to modify host processes [4]. Intense research is focused on identifying these proteins, called effectors, and to elucidate their complex functions. To date, over 80 *H. glycines* effectors have been identified and confirmed [5, 6], although many more remain to be discovered. Characterization of some known effectors has provided critical insights into the parasitic strategies of *H. glycines*. For example, these studies revealed that effectors are involved in a suite of functions, including defense suppression, plant hormone signaling alteration, cytoskeletal modification, and metabolic manipulation (reviewed by [7–10]). However, research has yet to provide a basic understanding of the molecular basis of virulence, i.e., the ability of some nematode populations to infect soybean plants with resistance genes, while other nematode populations are controlled by these resistance genes.

*H. glycines* populations are categorized into Hg types based on their virulence to a panel of soybean cultivars with differing resistance genetics [2, 11]. Based on the Hg type designation, growers can make informed decisions on soybean cultivar choice. To date, the Hg type designation can only be ascertained through time-consuming and expensive greenhouse experiments. However, once the genetic basis for virulence phenotypes has been explored, it is conceivable that molecular tests can be developed to make Hg type identification fast and reliable.

Resistant soybean cultivars are becoming less effective, as *H. glycines* populations alter their Hg type designation as a function of the soybean resistance genes to which the nematode population is exposed. In other words, when challenged with a resistant soybean cultivar for an extended duration, the surviving nematodes of an otherwise largely non-virulent *H. glycines* population will eventually shift towards a new Hg type that is virulent on resistant soybean cultivars [2]. It is unknown if this phenomenon solely relies on the selection of virulent genotypes already present within a given nematode population, or if *H. glycines* wields the power to diversify an existing effector portfolio to quickly infect resistant soybean cultivars. In addition, such genetic shifts appear to be distinct across populations with the same pathotype, indicating populations can independently acquire the ability to overcome host resistance [12]. Understanding these and other questions targeting the molecular basis of *H. glycines* virulence are critical for sustainable soybean production in a time when virulent nematodes are becoming more prevalent.

Scientists can finally start answering such questions, as we are presenting a near-complete genome assembly and extensive effector annotation of *H. glycines*, along with single-nucleotide polymorphisms (SNPs) associated with fifteen *H. glycines* populations of differing virulence phenotypes. PacBio long-reads were assembled and annotated into 738 contigs of 123 Mb containing 29,769 genes. The *H. glycines* genome has significant numbers of repeats (34% of the genome), tandem duplications (14.6 Mb), and horizontal gene transfer events (151 genes). Using this genome, we explored potential mechanisms for how effectors originate, duplicate, and diversify. Specifically, we found that effectors are frequently associated with tandem duplications, DNA transposons, and LTR retrotransposons. Additionally, we have leveraged RNA-seq data from pre-parasitic and parasitic nematodes and DNA sequencing across 15 *H. glycines* populations to further characterize effector expression and diversity.

## Results

### Genome assembly, annotation, and completeness

**Fig. 1:**
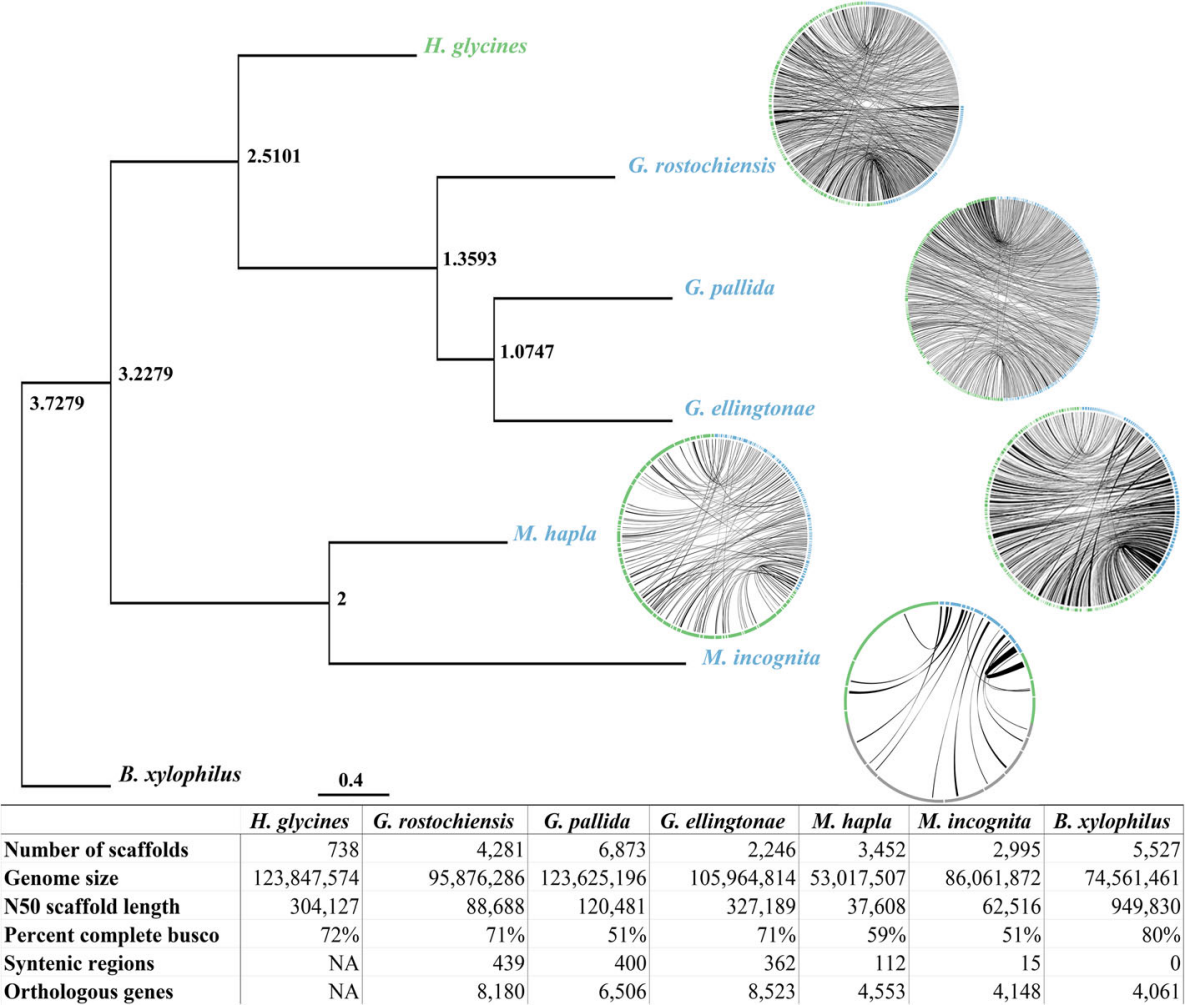
Phylogenetic relationships of species related to *H. glycines*. Phylogenetic tree of BUSCO genes with synteny representing the relatedness of each species. Synteny is inversely correlated with phylogenetic distance, as syntenic multiplicons decrease from hundreds in *Globodera* species, to zero in *B. xylophilus*, Green contigs denote *H. glycines*, while blue represent the respective related species. Node labels represent node ages. Pertinent comparative genome stats are found in the footer

*H. glycines* genomic DNA was extracted, and PacBio sequencing generated 2.4 million subreads with an average length of 7.6 kb corresponding to a coverage of 141× at an estimated genome size of 129 MB [13]. Due to the high level of heterozygosity of *H. glycines* populations, our early PacBio-only assemblies using Falcon and Falcon-Unzip resulted in an abundance of heterozygous contigs (haplotigs). Therefore, we reduced the heterozygosity of the original reads using a combination of Falcon, CAP3, and manual scaffolding of the assembly graph in Bandage. The final assembly was polished with Quiver and contains 738 contigs with an N50 of 304 kb and a total genomic content of 123,846,405 nucleotides (Fig. 1). We confirmed the assembly to be free of contamination using Blobtools (4.8.2) (Additional file 1: Figure S1) and validated for completeness by alignment of raw data: 88% of the RNA-seq [14] and 93% of the PacBio preads (Additional file 1: Table S1). In addition, approximately 72% of the 982 Nematoda-specific BUSCO genes are complete in the *H. glycines* genome, which is comparable to BUSCO scores in other *Tylenchida* genomes (Additional file 1: Table S2). Remarkably, only 56% of the BUSCO genes in *H. glycines* are single-copy, while 16% were duplicated, a statistic that is comparable to the allopolyploid root-knot nematode *Meloidogyne incognita* (Additional file 1: Table S2) [15–17]. Synteny diminished as phylogenetic relatedness declined (Fig. 1, Additional file 1: Figures S2-S6), supporting the established phylogeny alongside a phylogenetic tree (Fig. 1) derived from 651/982 single-copy BUSCO genes shared by at least three species among *H. glycines*, *Globodera pallida*, *Globodera ellingtonae*, *Globodera rostochiensis*, *Meloidogyne hapla*, *M. incognita*, and *Bursaphelenchus xylophilus*.

Gene annotations were performed using Braker on an unmasked assembly, as multiple known effector alignments were absent from predicted genes when the genome was masked (Additional file 1: Figure S7). While all known effectors are present in the assembly, the resulting gene count of 29,769 also includes many expressed repetitive elements (12,357). A variety of transcriptional sequencing was used as input for gene annotations, including 230 million RNA-seq reads from both pre-parasitic and parasitic J2 *H. glycines* nematodes [14], 34,041 iso-seq reads from early, middle and late life stages of both a virulent and an avirulent strain, and the entirety of the *H. glycines* ESTs in NCBI (35,796).

### Effector gene identification

**Fig. 2:**
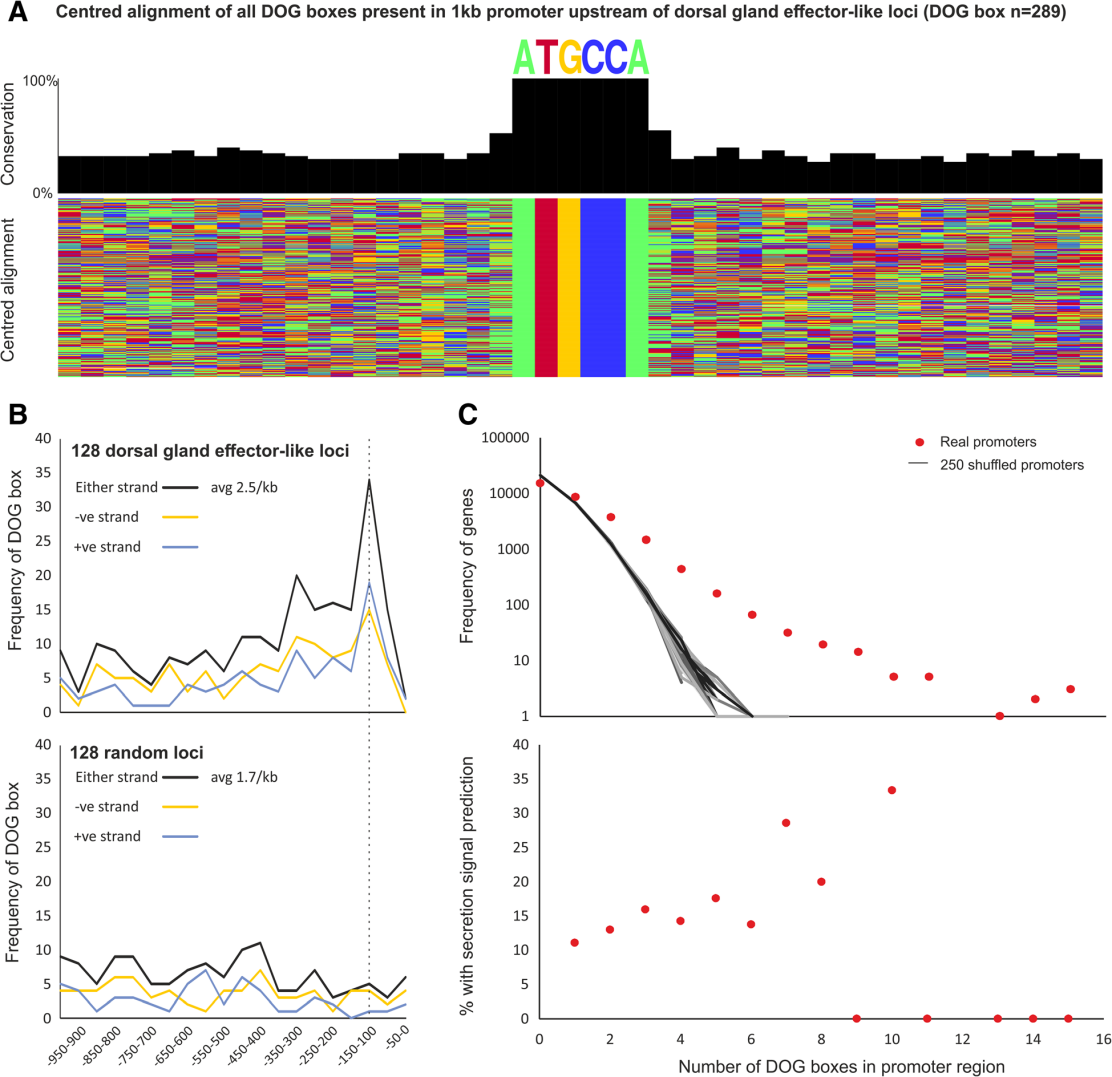
DOG boxes in dorsal gland effector-like loci. **a** Centered alignment of DOG boxes found in *H. glycines* promoters using HOMER. **b** DOG-box positional enrichment upstream of promoters in DOG-box genes and a lack of enrichment for 128 random gene promoters. **c** Increased frequency of DOG boxes in promoters in a small subset of genes compared to random promoters and likelihood of DOG box prediction of secretion signal peptide

Effector genes give rise to proteins that are secreted into the host to modify host cellular processes. Many effectors originate in the esophageal glands. Dorsal gland-expressed genes (DOGs) are mostly active during the later parasitic stages when syncytial development is initiated and progressing. In *Globodera* cyst nematode species, a putative regulatory promoter motif of dorsal gland cell expression, the DOG box, was recently identified [12]. To determine whether the regulation of dorsal gland cell expression in *Heterodera* species may be under similar control, we generated a non-redundant list of putative homologues of known dorsal gland effectors from cyst nematodes. This included all known dorsal gland effectors, a large family of recently characterized glutathione synthetase-like effectors [18], and all DOG-box associated effectors of *G. rostochiensis*. A total of 128 unique dorsal gland effector-like loci were identified in the genome, their promoter regions were extracted and compared to a random set of non-effector gene promoters using a non-biased differential motif discovery algorithm. Using this approach, a near-identical DOG box motif was identified (Fig. 2a), enriched on both strands of dorsal gland effector-like loci promoters approximately 100–150 bp upstream of the start codon (Fig. 2b). DOG box motifs occur at a greater frequency in promoters than expected by random, however their presence in a promoter is only a modest prediction of secretion (Fig. 2c). Taken together this suggests that the cis-regulatory elements controlling dorsal gland effector expression may be a conserved feature in cyst nematodes, predating at least the divergence of *Globodera* and *Heterodera* over 30 million years ago.

Given that DOG boxes are only present in some effector promoters, to identify a comprehensive repertoire of effectors we combined several methods and criteria. First, we aligned the 80 known *H. glycines* effector sequences to the genome using GMAP, identifying 121 putative effector genes. Second, the same 80 known effectors were subjected to motif discovery with MEME, identifying 24 motifs in 60/80 effectors (Additional file 1: Figure S8). One motif (motif 1) was a known signal peptide found in 10/60 effectors [19]. In addition, motifs 8, 12, and 18 were also consistently found at the N terminus in 7/60, 16/60, and 17/60 effectors, respectively. Because genes containing these motifs may also be effectors, the 24 motifs (Additional file 2: Data S1) were queried against the *H. glycines* predicted proteome using FIMO, revealing a set of 292 proteins with at least one effector-like motif. All three effector gene predictions were merged to produce a unique set of 431 effector-like genes. This gene set was used in downstream analyses exploring effector evolution. Of the 431 effector-like loci, 216 are predicted to encode a secretion signaling peptide and lack a transmembrane domain. While the remaining 215 effector-like loci may contain non-effectors, they were retained for downstream evolutionary analyses because they may represent genes with non-canonical secretion signals, “progenitor” housekeeping genes that gave rise to effectors (e.g. GS-like effectors [18], SPRY-SECs [20], etc.), or an effector graveyard.

**Fig. 3:**
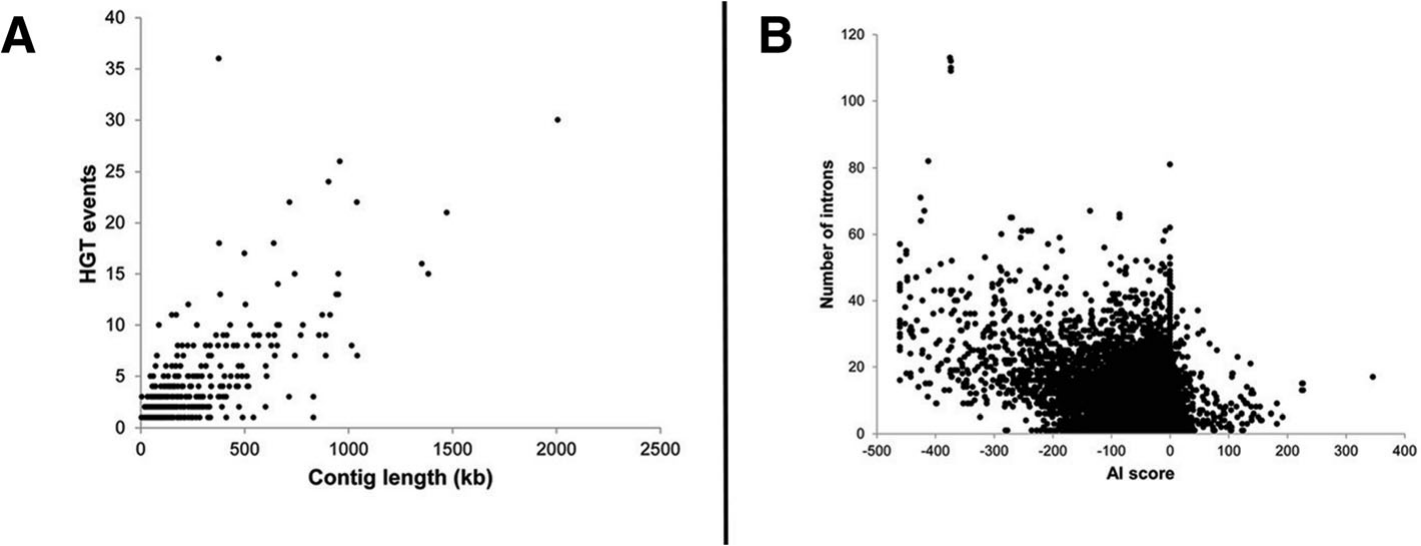
SNP density (a) and expression (b) of effector genes and gene copy number variants. Effectors found within repetitive regions were not significantly associated with SNP changes, although both effectors and secreted genes in any region of the genome were frequently up and downregulated in expression. Significance was calculated with Fisher’s exact tests in the GeneOverlap R package, and significance at <0.05 is *, <0.01 is **. Expression is represented as a log fold change of expression in a comparison of pre-parasitic and parasitic J2 nematodes

Horizontal gene transfer (HGT) was important for the evolution of parasitism in the root-knot and cyst nematodes [21–28]. To better understand the role of HGT in the evolution of effectors in *H. glycines*, we calculated an Alien Index (AI) for each transcript using a ratio of similarity to metazoan and non-metazoan sequences [29]. A total of 1678 putative HGT events (AI> 0) were observed in the predicted *H. glycines* proteome (Additional file 3: Data S2), distributed on 461 different contigs (Fig. 3a). This prediction includes 151 genes with strong HGT support (AI> 30) (Fig. 3b), 82 genes previously identified in closely related nematodes (Additional file 1: Table S3), and 107 putative HGT reported here for the first time in plant parasitic nematodes (Additional file 4: Data S3). The number of introns was significantly reduced in genes with AI> 0 (6.8 vs 9.7, *p* < 0.001, Student’s t-test) (Fig. 3b), further supporting a non-metazoan origin. Among these, the highest E-values were of bacterial, fungal, or plant origin for 70.8% (114/161), 19.3, and 9.9%, respectively (Additional file 3: Data S2). Interestingly, only 7/151 high confidence HGT genes were co-identified as one of the 431 effector-like loci.

### Genomic insights into the mechanisms of effector duplication and diversification

The tandem duplication (TD) of genes in pathogen genomes is a common evolutionary response to the arms race between pathogen and host as a means to avoid/overcome host resistance [30]. To identify the role tandem duplications play in the duplication virulence genes, we implemented RedTandem to survey the *H. glycines* genome. We determined that a total of 18.7 MB of the genome is duplicated with a total of 20,577 duplications in the genome. While most individual duplications were small, the average tandem duplication size was 909 bp. We verified that tandem duplications were not assembly artifacts by aligning the PacBio preads to the genome and confirmed that the larger than average tandem duplications (4410/4241) were spanned by PacBio preads across > 90% of tandem duplication length. The density of genes in the tandemly duplicated regions is higher than in non-duplicated regions of the genome: 6730/18.7 MB (~ 360 genes/MB) vs 23,039/105.2 MB (~ 219 genes/MB), and thus contributes to one fifth of the total gene count in the *H. glycines* genome. The largest groups of orthologous genes found in tandem duplications (881/3940 genes) were annotated with BLAST to the NCBI non-redundant (NR) database, revealing that the 38 largest clusters of duplicated genes were frequently transposable element genes, effector/gland-expressed genes, or BTB/POZ domain-containing genes (Additional file 1: Figure S9). Both effector-like loci (136/431; 36%) and HGT genes (38/151; 25%) were duplicated in the tandem duplications. Of effectors that were orthologous in the tandemly duplicated orthologs, Hgg-20 (144), 4D06 (11) and 2D01 (11) were the most frequent, while RAN-binding proteins formed the largest cluster of HGT genes (Additional file 1: Figure S9).

**Fig. 4:**
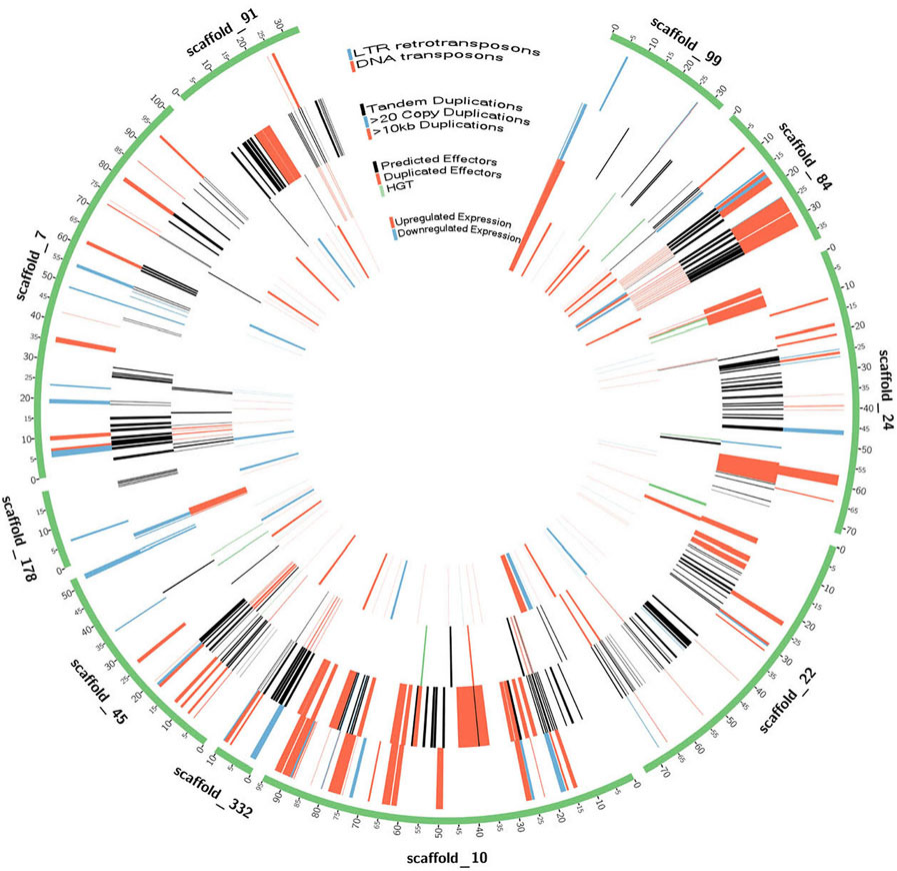
Most highly duplicated scaffolds in the *H*. *glycines* genome colocalizing with effector genes. Scaffold ticks signify 50kb increments along scaffolds (green), High correlations were found between regions containing transposon elements and tandem duplications. Effectors were frequently duplicated within these regions. HGT events were evenly distributed across scaffolds, while upregulated and downregulated expression was frequently associated with variable up and down regulation of expression

To investigate whether transposons were associated with the expansion of effector genes, we created confident transposon and retrotransposon models using data co-integrated from RepeatModeler, LTR finder, and Inverted Repeat Finder (see methods). One-third of the *H. glycines* genome was considered repetitive by RepeatModeler (32%, 39 Mb) with the largest classified types being DNA transposons (7.53%), LTR elements (2.92%), LINEs (1.83%) and SINEs (0.04%) (Additional file 1: Table S4). To identify full-length DNA transposons and LTR retrotransposons, Inverted Repeat Finder (3.07) and LTR Finder (1.0.5) were used to identify terminal inverted repeats (TIRs) and LTRs, respectively. The genomic co-localization of RepeatModeler repeats and inverted repeats led to the identification of 1075 DNA transposons with a mean size of 6.6 kb and encompassing 1915 genes (Fig. 4). Similarly, the overlap of RepeatModeler repeats and LTR Finder repeats identified 592 LTR retrotransposons with 8.1 kb mean size and encompassing 1401 genes (Fig. 4). Among the genes found within DNA or retro-transposon borders, 58/1915 and 22/1401 were predicted effectors, respectively. Indeed, many transposon-associated genes had effector-like functions (Additional file 1: Figure S9), as seen in a tandemly duplicated transposable element carrying known effectors and effector-like genes (Additional file 1: Figure S10). Transposon-mediated duplication is not specific to effectors, as evidenced by 14 duplicated HGT RAN-binding proteins. To obtain a measure of duplications associated with transposons, Bedtools intersect was used to identify transposon-associated gene overlap with tandemly duplicated genes. Of the 6730 genes contained in tandem duplications, 969 and 656 were contained in DNA and LTR transposons, respectively.

**Fig. 5:**
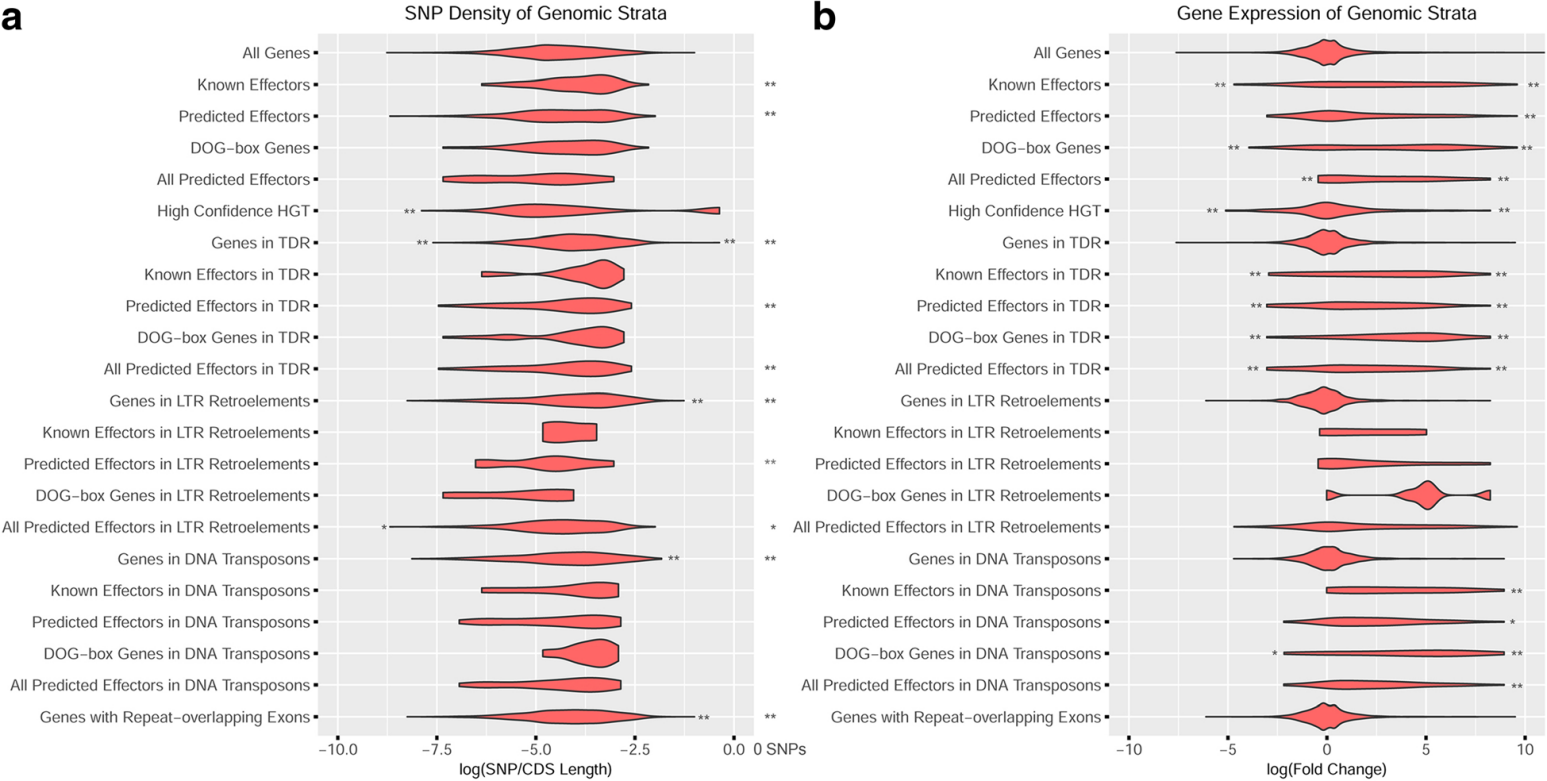
SNP density and expression of effector genes and gene categories. Effectors found within repetitive regions were not significantly associated with SNP changes, although both effectors and secreted genes in any region of the genome were frequently up and downregulated in expression. Significance was calculated with Fisher’s exact tests in the GeneOverlap R package, and significance at < 0.05 is *, < 0.01 is **. Statistical comparisons were peformed between gene categories and high SNP density, low SNP density, zero SNPs, high expression, and low expression. Expression is represented as a log fold change of expression in a comparison of pre-parasitic and parasitic J2 nematodes

Another possible mechanism by which *H. glycines* could overcome soybean resistance is through changes in coding sequences that result in differences among closely related effectors. Therefore, identifying SNPs in effector genes may reveal mutations associated with effector diversification. Using GATK best practices [31], 1,619,134 SNPs were identified from 15 bulked, pooled DNA preparations from isolate populations of virulent and avirulent *H. glycines* lines. To better understand population-level dynamics SNP-Relate was used to create a PCA plot, and as expected, populations primarily grouped by their original ancestral population but also by selection pressure on resistant cultivars (Additional file 1: Figure S11). The SNP density for each gene was determined by dividing SNP frequency by CDS length, and Fisher’s exact tests with the GeneOverlap R package were used to identify significant associations with genes in the 10th and 90th percentile of SNP density (Fig. 5, Additional file 5: Table S5). SNP-dense genes were significantly enriched for genes found in tandem duplications, DNA transposons, LTR retrotransposons, and any gene with exon-overlapping repeats. While mutations are present in effectors, effector genes were not associated with high SNP density, although the lack of unique reads in highly duplicated regions may be responsible. Supporting this hypothesis, genes and effectors found in tandem duplications, DNA transposons, and LTR retrotransposons significantly overlapped with the 4613 genes lacking SNPs, and thus unique sequence reads (Fig. 5, Additional file 5: Table S5).

### Genomic structures associated with gene expression change in *H. glycines*

To assess the importance of genes affected by duplications, repeat-association, and SNP density, we utilized gene expression from second-stage juveniles of *H. glycines* population PA3 before and after root infection of a resistant and susceptible soybean cultivar (SRP122521). Genes differentially up and downregulated after infection were identified using DESEQ with a q-value cutoff of 1e-8, revealing 1211 and 568 genes with significant up and down regulation, respectively. To associate differential expression with effectors and other gene categories, significant associations were identified using the GeneOverlap R package (Fig. 5, Additional file 5: Table S5). As expected, many of the predicted effectors were significantly upregulated upon infection, a trend that continued with putative effectors found in DNA transposons and tandem duplications. In contrast, the only significantly upregulated gene categories not directly associated with predicted effectors were secreted genes and genes associated with an effector-associated repeat (Family-976 repeat).

However, since virulence genes have a limited span of use before host immunity is developed, the expression of a recognized effector may hinder survival, thus finding effectors with reduced gene expression is not surprising. Generally, genes associated with tandem duplications, HGT, and transposons had similar distributions of expression as genes that were non-associated, yet effectors found in tandem duplications and DNA transposons were significantly enriched for genes with high and low expression (Fig. 5). This high and low expression trend in effectors was also apparent in secreted genes at a higher significance, indicating that many potential effectors remain elusive to detection.

## Discussion

To overcome the expected assembly problems associated with high-levels of repetitive DNA and to reveal the evolutionary means behind the rapid evolution and population shifts in *H. glycines*, we used long-read technology to assemble a genome from a heterogenous population of individuals. Several analyses confirmed a high level of genome completeness with ~ 88% of the RNA-Seq aligning, 93% of preads aligning, and zero contaminating scaffolds (Additional file 1: Table S1, Figure S1). While percentages of missing BUSCO [32] genes were high, BUSCO genes were 72% complete, ranking *H. glycines* the best among sequenced genomes in the cyst and knot-nematode clades (Fig. 1, Additional file 1: Table S2). Some level of artifactual duplication may be present in the genome, with BUSCO gene duplication being highest among the species analyzed. However, only 79/349 duplicated BUSCO genes are found in tandem duplications, indicating that duplication or heterozygous contigs may be present elsewhere in the genome. With a goal-oriented approach of capturing all genic variation in the genome, we sequenced a population of multiple individuals. We therefore assembled a chimera of individuals, with some duplicated genes originating from single variants in the population. However, even when considering that nearly nine thousand genes could be attributed to repetitive elements and tandem duplications, the gene frequency (20,830) and exon statistics of *H. glycines* are elevated in relation to sister Tylenchida species.

Because plant parasitism has independently arisen three times in Nematoda, and because it is thought that HGT plays a crucial role in the nematodes’ adaptation to this lifestyle [25, 33], we investigated the potential role HGT may have in *H. glycines*. Almost all previously identified HGT in plant-parasitic nematodes were also found in *H. glycines* (*n* = 82) (Additional file 1: Table S3) [34]. Genes with strong AI (> 30) were mainly hydrolases, transferases, oxidoreductases or transporters (Additional file 3: Data S2). Of interest were genes originating from bacteria or fungi, but lacking BLAST hits to Metazoan species (highlighted blue in Additional file 4: Data S3). Among these is a gene coding for an Inosine-uridine preferring nucleoside hydrolase (Hetgly.000009703; AI = 101.2), an enzyme essential for parasitism in many plant-pathogenic bacteria and trypanosomes [35]. A candidate oomycete RxLR effector [36] was also identified in the genome (Hetgly.000002962, Hetgly.000002964 and Hetgly.000002966; AI up to 42.2). Besides being necessary for successful infection, RxLR effectors are also avirulence genes in some species, including the soybean pathogen *Phytophthora sojae* [37]. The *H. glycines* genome is also host to a putative HGT gene (Hetgly.000001822 and Hetgly.000022293; AI up to 55.3) that has been characterized as *a G. pallida* effector (Gp-FAR-1) involved in plant defense evasion by binding plant defense compounds [38]. Thus, horizontal gene transfer appears to contribute to the evolution of *H. glycines* virulence as well as to the ancestral development of parasitism in plant-parasitic nematodes [33, 39–41].

Although HGT is more common among nematodes and arthropods than other animals [42], there are many documented cases of gene duplication leading to evolutionary novelty and phenotypic adaptation across metazoans [43, 44]. With over a fifth of the genes in the *H. glycines* genome found in tandem duplications, characterizing the largest clusters of orthologous gene families in tandem duplications provides relevant information for identifying genes related to parasitism, adaptation, and virulence. A functional assessment of the 38 largest clusters of tandemly duplicated orthologues were largely transposon-associated proteins or proteins related to effectors, indicating that transposons have a role in duplicating effector genes (Additional file 1: Figures S9, S10). Because many of the LTRs and TIRs were nested, the frequent rearrangements of nested clusters of transposons [45] could be attributed to effector exon shuffling [46]. While genes in duplicated regions of the genome were significantly associated with high SNP density (Fig. 5a), putative effectors were not. While it is known that genes in duplicated regions pave a way for evolutionary novelty [43, 44], the lack of high SNP density for effectors in duplicated regions may represent low sequencing depth or the recent duplication of these loci. While significant effector mutations could not be found in these regions, these effectors were some of the most highly upregulated and downregulated genes upon infection (Fig. 5b).

## Conclusions

The *H. glycines* genome assembly and annotation provides a glimpse into host and parasite interplay through the characterization of known and predicted effector genes. This relationship is further unraveled through the characterization of tandem duplications, horizontal gene transfers, transposon hitchhiking, promotor regulatory element identification, alternative splicing, SNP density, and gene expression. The generation of these genomic resources will facilitate a greater understanding of the host-parasite relationship by revealing genes involved in creating and maintaining a functional feeding site. Thus, the genomic analysis of the *H. glycines* genome is an important advance in the pathway to generating new forms of resistance and control measures against *H. glycines*.

## Methods

### Nematode culture and DNA/RNA isolation

*H. glycines* inbred population TN10, Hg type 1.2.6.7, was grown on susceptible soybean cultivar Williams 82 in a greenhouse at Iowa State University. A starting culture of approximately 10,000 eggs from Dr. Kris Lambert, University of Illinois, was bulked for four generations on Williams 82 soybeans grown in a 2:1 mixture of steam pasteurized sand:field soil in 8″ clay pots, with approximately 16 h daylight at 27 °C. Genomic DNA was extracted from approximately 100,000 eggs in a subset of third generation cysts. Egg extraction was performed with standard nematological protocols [47], eggs washed 3 times in sterile 10 mM MES buffered water, and pelleted before flash freezing in liquid nitrogen.

Genomic DNA was isolated using the MasterPure Complete DNA Purification Kit (Epicentre) with the following modifications: Frozen nematode eggs were resuspended in 300 ul of tissue and cell lysis solution, and immediately placed in a small precooled mortar, where the nematode solution refroze and was finely ground. The mortar was then placed in a 50 °C-water bath for 30 min, then transferred to 500 ul PCR tubes with 1 ul of proteinase K, and incubated at 65 °C for 15 min, inverting every 5 min. Genomic DNA was resuspended in 30 ul of RNAse/DNase free water, quantified via nanodrop, and inspected with an 0.8% agarose gel at 40 V for 1 h. Two 20 kb insert libraries were generated and sequenced on 20 PacBio flow cells at the National Center for Genome Resources in Santa Fe, NM (SRR5397387 – SRR5397406).

Fifteen *H. glycines* populations were chosen based on Hg-type diversity and were biotyped to ensure identity (TN22, TN8, TN7, TN15, TN1, TN21, TN19, LY1, OP50, OP20, OP25, TN16, PA3, G3). Information on the selection and Hg-types of these lines is available in Additional file 6: Table S6. Genomic DNA from approximately 100,000 eggs for each population was extracted as described previously, and 500 bp libraries were sequenced on an Illumina HiSeq 2500 at 100PE (SRR5422809 – SRR5422824).

Six life stages were isolated for both PA3 and TN19 *H. glycines* populations: eggs, pre-parasitic second-stage juveniles (J2), parasitic J2, third-stage juveniles (J3), fourth stage juveniles (J4) and adult females. Parasitic J2 were isolated, followed by isolations of J3, J4, and adult females at 3, 8, 15, and 24 days post-infection via a combination of root maceration, sieving and sucrose floatation, using standard nematological methods [47]. Total RNA was extracted with the Exiqon miRCURY RNA Isolation Kit (Catalog #300112). RNA was combined to form three pools for each population, corresponding to early (egg and pre-parasitic J2), middle (parasitic J2 and J3) and late (J4 females and early adult females) developmental stages. The IsoSeq data were used to improve the annotation (see below) (SAMN08541516-SAMN08541521).

### Genome assembly

A PacBio subreads assembly was generated with Falcon to correct subreads into consensus preads (error corrected reads), followed by contig assembly. An alternative approach using only transcript containing preads was helpful in solving heterozygosity and population problems. Transcripts were aligned to preads using Gmap [48] under default parameters, and a pool of preads for each unique transcript was assembled using CAP3 [49] under default parameters. The longest assembled contigs and all unassembled preads were retained and read/contig redundancy was removed with sort and uniq. New FASTA headers were generated using nanocorrect-preprocess.pl [https://github.com/jts/nanocorrect/blob/master/nanocorrect-preprocess.pl], and sequences were then assembled with Falcon with default settings into 2692 contigs (supp file *H. glycines*.cfg). Falcon output was converted to Fastg with Falcon2Fastg [https://github.com/md5sam/Falcon2Fastg], and longer scaffolds were created with Bandage [50] using multiple criteria. 1) The longest path was chosen and ended with an absence of edges. 2) If the orientation of an interior contig was disputed, one set of edges was deleted to extend the scaffold. 3) The shortest path through difficult repetitive subgraphs was chosen.

Intragenomic synteny was used to remove clonal haplotigs [51, 52] (synteny as below). When synteny was identified between two contigs/scaffolds, if a longer 3′ or 5′ fragment could be made, then the ends of each contig/scaffold were exchanged at the syntenic/non-syntenic juncture. All remaining duplicate scaffolds retaining synteny were truncated or removed from the assembly, and followed by a BWA [53] self-alignment to remove redundant repetitive scaffolds at a 90% identity threshold.

### Genome quality control

Multiple measures were taken to assess genome assembly quality, including a default BLASR [54] alignment of PacBio subreads, preads, and ccsreads resulting in alignment percentages at 88.7, 93.3, 90.1%, respectively (Additional file 1: Table S1). Using default settings, Gmap and Hisat2 (2.0.3) mapped 86.4% percent of a transcriptome assembly and ~ 88% of the five RNA-seq libraries, respectively (Additional file 1: Table S1). Genome completeness was assessed with BUSCO [32] at 71.9%. An absence of contamination was found with Blobtools (4.8.2) [55] using MegaBLAST (2.2.30+) to the NCBI nt database, accessed 02/02/17, at a 1-e5 e-value. See Additional file 7 for more detail.

### Genome annotation

To account for the high proportion of noncanonical splicing in nematodes [12], Braker [56] was used to predict genes using Hisat2 (2.0.3) [57] raw RNA-Seq alignments of ~ 230 million 100 bp PE RNA-Seq reads [14] and GMAP [58] alignments of IsoSeq reads, and all EST sequences from NCBI. Because gene models were greatly influenced by repeat masking, three differentially repeat-masked genomes were used for gene prediction: unmasked, all masked, and all except simple repeats masked (see supp table RNASEQ mapping in excel). All protein isoforms were annotated with Interproscan [59, 60] in BlastGO [61], and with BLAST [62] to Swiss-prot [63] and Uniref [64] at e-value 1e-5.

### Repeat prediction

Repetitive elements in the *H. glycines* genome were classified into families with five rounds of RepeatModeler (1.0.8) [65] at default settings, followed by genome masking with RepeatMasker [66] at default settings. Inverted Repeat Finder (3.07) and LTR Finder (1.0.5) were used at default settings to define the border of a TE only when overlapping RepeatModeler repeats were present. Supplemental helitron prediction was done with HelitronScanner [67] under default settings.

### Promoter analyses

To determine to what extent cyst nematodes use common mechanisms for dorsal gland effector regulation, we screened sequences previously associated with the DOG box in other genera against the *H. glycines* genome. The *G. rostochiensis* DOG-effectors [12] were used as queries in BLASTp to identify DOG-effector-like loci in the predicted proteome of *H. glycines*. The most similar sequence was retrieved if it satisfied two criteria, an e-value <1e-10 and the protein encoded a signal peptide for secretion (78 unique *H. glycines* loci). Using the same approach, 94 genes similar to other published dorsal gland expressed effectors (58) were identified [6] and combined with the DOG-effector-like list to a non-redundant 128 loci. Given the nature of these two criteria, not all sequences in this list will be effectors and not all effectors will be in this list, nevertheless, it will contain a sufficient number to determine whether the DOG box is conserved in *H. glycines*. A 500 bp region 5′ of the ATG start codon, termed the promoter region, was extracted from these 128 loci and used for motif enrichment analysis using HOMER [68], as previously described [12]. DOG-box positional enrichment was calculated using FIMO web server [69] and predictive power calculated using custom python scripts.

### Effector prediction

At default settings Gmap [58] was used to align 80 previously identified effectors to the genome [5, 6, 70, 71]. Conserved protein motifs in effectors were identified with MEME: -nmotifs 24, −minsites 5, −minw 7, −maxw 300, and zoops (zero or one per sequence) [72]. These motifs were used as FIMO queries to search the inferred *H. glycines* proteome [72].

### Synteny

The genome, gff, and peptide sequences for *C. elegans* (WBcel235), *G. pallida* [73], and *M. hapla* [74] were downloaded from WormBase [75]. The genome and gff of *G. rostochiensis* [12] was downloaded from NCBI. The *G. ellingtonae* genome was also downloaded from NCBI [76], but gene models were unavailable, thus gene models for *G. ellingtonae* were called with Braker using RNA-seq reads from SRR3162514, as described earlier.

Fastp and global alignments with Opscan (0.1) [77] were used to calculate orthologous gene families between *H. glycines* and *C. elegans* [78]*, G. pallida* [73]*, G. ellingtonae* [76]*, G. rostochiensis* [12]*, M. hapla* [79]*,* and *M. incognita* [15]. All alternatively spliced variants and all possible multi-family genes were considered (-C, −b, −Q).

To infer synteny, iAdHoRe 3.0.01 [80] was used with prob_cutoff = 0.001, level 2 multiplicons only, gap_size = 15, cluster_gap = 20, q_value = 0.9, and a minimum of 3 anchor points. Syntenic regions are displayed using Circos (0.69.2) [81].

### Phylogenetics

Predicted protein sequences from the aforementioned nematode genomes (excluding *C. elegans*) were scanned with BUSCO 2.0 [32] for 982 proteins conserved in *nematoda_odb9*. 651 proteins were found in at least 3 species and aligned with Prank [82] in Guidance [83] at default parameters. Maximum likelihood gene trees were computed using RAxML [84] with 1000 bootstraps and PROTGAMMAAUTO for model selection. Astral [85] at default settings was used to prepare a coalescent-based species tree.

### Tandem duplication

With default settings, ReDtandem.pl was used to identify tandem duplications in the genome [86]. Tandem duplicate orthologous genes were identified using a self-BLASTp to predicted proteins with 50% query length and 90% identity [62]. To annotate clusters of orthologous genes, groups of highly connected nodes or entire clusters were concatenated and queried with BLASTp to the NCBI NR database [87].

### SNP density and PCA analysis of fifteen *H. glycines* populations

Raw sequences from fifteen populations of *H. glycines* nematodes were quality checked with FastQC [88]. Virulence for each *H. glycines* population are available in Additional file 6: Table S6. Reads were aligned to the *H. glycines* genome using default parameters in BWA-MEM [53]. The BAM files were sorted, cleaned, marked for duplicates, read groups were added and SNP/Indel realignment were performed prior to calling SNPs and Indels with GATK. Custom Bash scripts were used to convert the vcf file into a gff for use with Bedtools (2.2.6) to identify SNP and exon overlap [89]. The density of SNPs was calculated by dividing the number of SNPs/CDS length (bp). Phasing and imputing SNPs with Beagle 4.1 [90, 91] followed by a PCA analysis of SNPs vs Hg-type virulence using SNPRelate (1.12.2) [92].

### RNA-seq expression

RNA-seq reads were obtained from NCBI SRA accession SRP122521. Briefly, SCN inbred population PA3 was grown on soybean cultivar Williams 82 or EXF63. Pre-parasitic second-stage juveniles and parasitic second stage juveniles were isolated from roots of resistant and susceptible cultivars at 5 days post-inoculation [14]. 100 bp PE reads were aligned to the genome using default settings with HiSat2 [57]. Read counts were calculated using default settings with FeatureCounts from the Subread package [93], followed by Deseq2 [94] at default settings to determine log-fold change between the pre-parasitic samples (2 × ppJ2_PA3) and parasitic J2 samples (2 × pJ2_s63, pJ2_race3_Forrest).

### Alternative splicing

The analysis of the global changes and effector specific effects in alternative splicing landscape was assessed following a recent de novo transcriptomics analysis of the *H. glycines* nematode effectors [14] Transcriptome annotation was constructed using 230 million RNA-Seq reads from both pre-parasitic and parasitic J2 *H. glycines* [14]*,* 34,041 iso-seq reads from three life stages of both a virulent and an avirulent strain, and *H. glycines* ESTs in NCBI (35,796). Specifically, using a standard alternative splicing analysis pipeline [95], 230 million reads from both pre-parasitic and parasitic J2 *H. glycines* [14] were preprocessed with Trimmomatic [96], aligned with Tophat 2.1.1 [97]*,* and quantified with Cufflinks 2.2.1 [98], followed by conversion of FPKM to TPM [99], and patterns assessment with IsoformSwitchAnalyzerR [100]. For the 80 previously identified effectors [6, 70, 71], the changes in the functional domain architectures between specific alternatively spliced isoforms are determined using InterPro domain annotation server with a focus on Pfam domains [101].

## Additional files

Additional file 1: **Figure S1.** Contamination check with Blobtools. Circles represent scaffolds, while their colors represent different Phyla. All putative contaminating scaffolds are false-positive and have *H. glycines* origins. The one outlier represents the mitochondrial scaffold, which was misassembled and collapsed to appropriate size. **Table S1.** Rates of read alignment to the genome for PacBio reads, RNA-seq, and Trinity transcripts. **Table S2.** Busco genes found in Complete, Single-copy, Duplicated, Fragmented, and Missing categories for assembled genomes in the Tylenchida. **Figure S2.** *Globodera rostchiensis* synteny. 439 syntenic regions were identified between *G. rostochiensis* and *H. glycines*. Green contigs are *H. glycines*, while blue contigs are *G. rostochiensis*. **Figure S3.** *Globodera pallida* synteny. 341 syntenic regions were identified between *G. pallida* and *H. glycines*. Green contigs are *H. glycines*, while blue contigs are *G. pallida*. **Figure S4.** *Globodera ellingtonae* synteny. 362 syntenic regions were identified between *G. ellingtonae* and *H. glycines*. Green contigs are *H. glycines*, while blue contigs are *G. ellingtonae*. **Figure S5.** *Meloidogyne hapla* synteny. 112 syntenic regions were identified between *M. hapla* and *H. glycines*. Green contigs are *H. glycines*, while blue contigs are *M. hapla*. **Figure S6.** *Meloidogyne incognita* synteny. 15 syntenic regions were identified between *M. incognita* and *H. glycines*. Green contigs are *H. glycines*, while blue contigs are *M. incognita*. **Figure S7.** Repeatmodeler contig alignments overlapping effector alignments in the genome. Three separate examples, with the top track representing final gene models, middle representing Repeatmodeler/Repeatmasker contig alignments, and the lower track representing known effector alignments. **Figure S8.** Motif analysis of effector sequences. The 80 known effector proteins were subjected to a MEME analysis, and motifs identified in 61 effector proteins implemented with FIMO to find effector candidates in the genome. **Table S3.** *H. glycines* genes previously shown to be acquired by horizontal gene transfer in closely related plant-parasitic nematodes. **Figure S9.** Network of interrelated tandemly duplicated genes. Connections indicate protein similarity, while the text represents the BLAST hits to NR for the three most highly connected nodes in each subnetwork (hexagons). **Table S4.** Repeatmodeler/Repeatmasker repeats identified in the *H. glycines* genome. **Figure S10.** Colocalized transposons, tandem duplications, and effectors. **A.** JBrowse display of scaffold_345, showing four tracks: Gene annotations, DNA transposons, 80 known effector alignments, and tandem duplications. The large transposon colocalizes with five tandem duplications, four of the 80 known effectors, and four genes annotated as effectors. **B.** A highly similar transposon on scaffold_97 with the same effector types present within and tandem duplications nearby. **Figure S11.** Principal components analysis of SNPs from 15 populations of *H. glycines* nematodes. Colors represent Hg-type, the capability to reproduce to a certain threshold on seven soybean cultivars. The labels by each circle represent the names of each population. **Figure S12.** Alternative splicing changes in isoforms for all genes in the genome. All isoforms containing an ORF in the transcriptome were analyzed for three biological groups, and all pairwise comparisons were considered to show the changes in transcript structures caused by alternative splicing. AS isoform structures are characterized by three types of annotations: intron retention, NMD (nonmediated decay), and effect on ORF. **Figure S13.** Alternative splicing changes in isoforms for all effectors in the genome. Effector isoforms containing an ORF in the transcriptome were analyzed for three biological groups, and all pairwise comparisons were considered to show the changes in transcript structures caused by alternative splicing. AS isoform structures are characterized by three types of annotations: intron retention, NMD (nonmediated decay), and effect on ORF. (DOCX 6288 kb)

Additional file 2: **Data S1.** MEME motifs annotated with BLAST annotations to NR. Annotated MEME motifs from effector motif-finding. (TXT 1 kb)

Additional file 3: **Data S2.** All horizontal gene transfer events. All putative horizontal gene transfer events above an alien index of zero. (XLSX 398 kb)

Additional file 4: **Data S3.** Horizontal gene transfer events novel to *H. glycines.* Horizontal gene transfer events not previously reported in other plant parasitic nematodes. (XLSX 19 kb)

Additional file 5: **Table S5.** Significance tests for gene expression and snp density (XLSX 15 kb)

Additional file 6: **Table S6.** Nematode isotypes and selection. Hg-types, selection, and heritage of nematodes using in sequencing. (XLSX 12 kb)

Additional file 7: Supporting analyses text. (DOCX 22 kb)

## Abbreviations

AI: Alien index
BTB: BR-C, ttk, and bab domain containing
CDS: Coding region
DNA: Deoxyribonucleic acid
DOG: Dorsal expressed gene
EST: Expressed sequence tag
GS-like: Glutathione synthase-like
HGT: Horizontal gene transfer
LINE: Long interspersed nuclear element
LTR: Long terminal repeat
NR: Non-redundant protein database
PCA: Principal components analysis
POZ: Pox virus and zinc finger domain
RAN: RAs-related Nuclear protein
RNA: Ribonucleic acid
SCN: Soybean cyst nematode
SINE: Short interspersed nuclear element
SNP: Single nucleotide polymorphism
SPRY-SEC: Secreted SPRY domain-containing protein
TD: Tandem duplication
TIR: Terminal inverted repeat.
