# Correction: The genome of the soybean cyst nematode (*Heterodera glycines*) reveals complex patterns of duplications involved in the evolution of parasitism genes

**DOI:** 10.1186/s12864-024-10466-0

**Published:** 2024-05-31

**Authors:** Rick Masonbrink, Tom R. Maier, Usha Muppirala, Arun S. Seetharam, Etienne Lord, Parijat S. Juvale, Jeremy Schmutz, Nathan T. Johnson, Dmitry Korkin, Melissa G. Mitchum, Benjamin Mimee, Sebastian Eves-van den Akker, Matthew Hudson, Andrew J. Severin, Thomas J. Baum

**Affiliations:** 1 Department of Plant Pathology, Iowa State University, Ames, IA USA; 2 Genome Informatics Facility, Iowa State University, Ames, IA USA; 3 Agriculture and Agri-Food Canada, Saint-Jean-sur-Richelieu, QC Canada; 4 Department of Energy, Joint Genome Institute, Walnut Creek, CA USA; 5 HudsonAlpha Institute for Biotechnology, Huntsville, AL USA; 6 Bioinformatics and Computational Biology Program, Worcester Polytechnic Institute, Worcester, MA USA; 7 Department of Computer Science, Worcester Polytechnic Institute, Worcester, MA USA; 8 Division of Plant Sciences, University of Missouri, Columbia, MO USA; 9 Department of Plant Sciences, University of Cambridge, Cambridge, UK; 10 Department of Crop Sciences, University of Illinois, Urbana, IL USA

**Correction**: ***BMC Genomics*** **20, 119 (2019)**

10.1186/s12864-019-5485-8

Following publication of the original article it was reported that the wrong Genbank accession numbers are reported in the ‘Availability of data and materials’ declaration. Under the Availability of data and materials section, SRR6782833 - SRR6782842 should be SRR12508204 - SRR12508212.

