# Supplementary material for: The genome of the soybean cyst nematode (Heterodera glycines) reveals complex patterns of duplications involved in the evolution of parasitism genes
Source: BMC Genomics. 2019 Feb 7;20:119. doi: 10.1186/s12864-019-5485-8 (PMC6367775; doi:10.1186/s12864-019-5485-8)

Figure S1
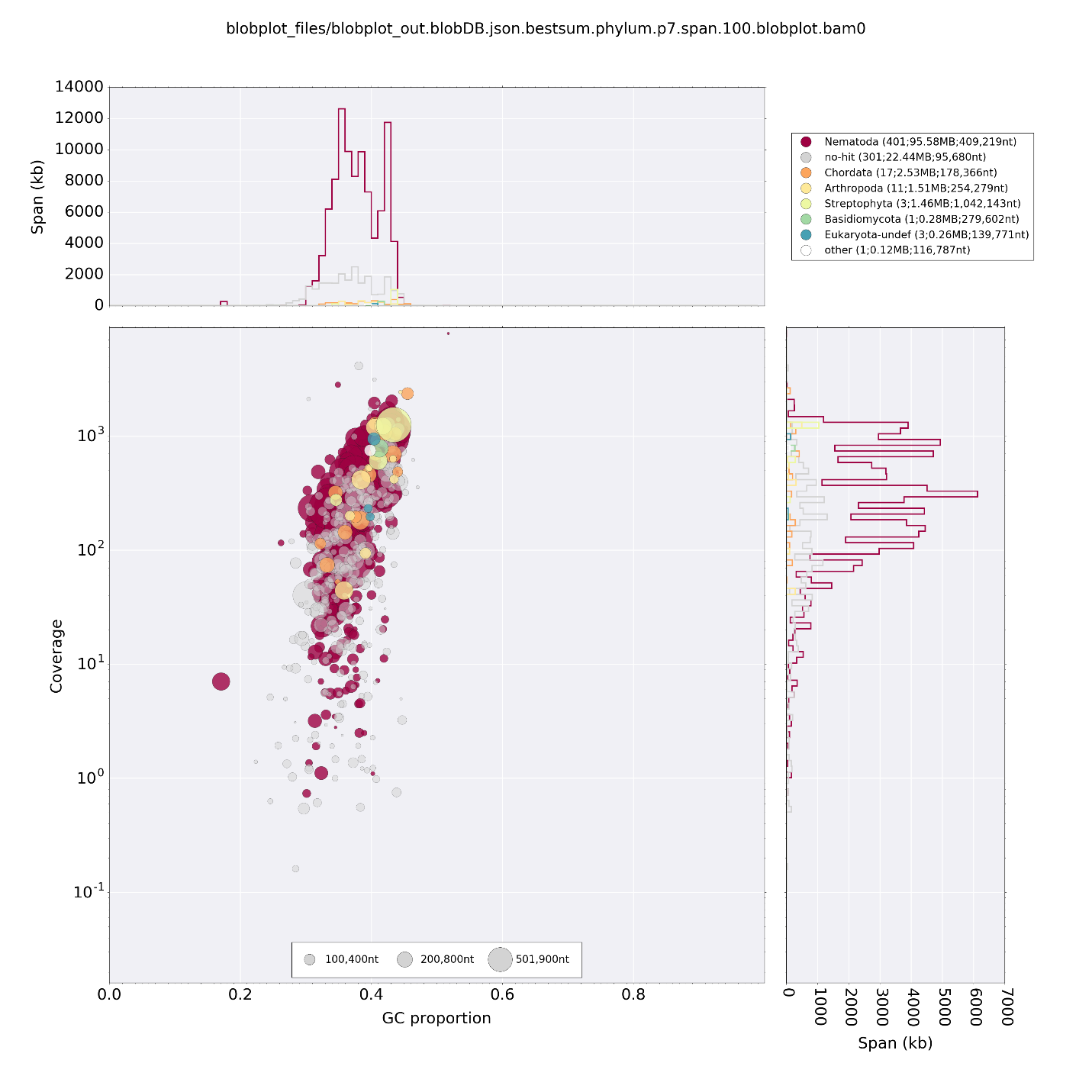


Figure S2


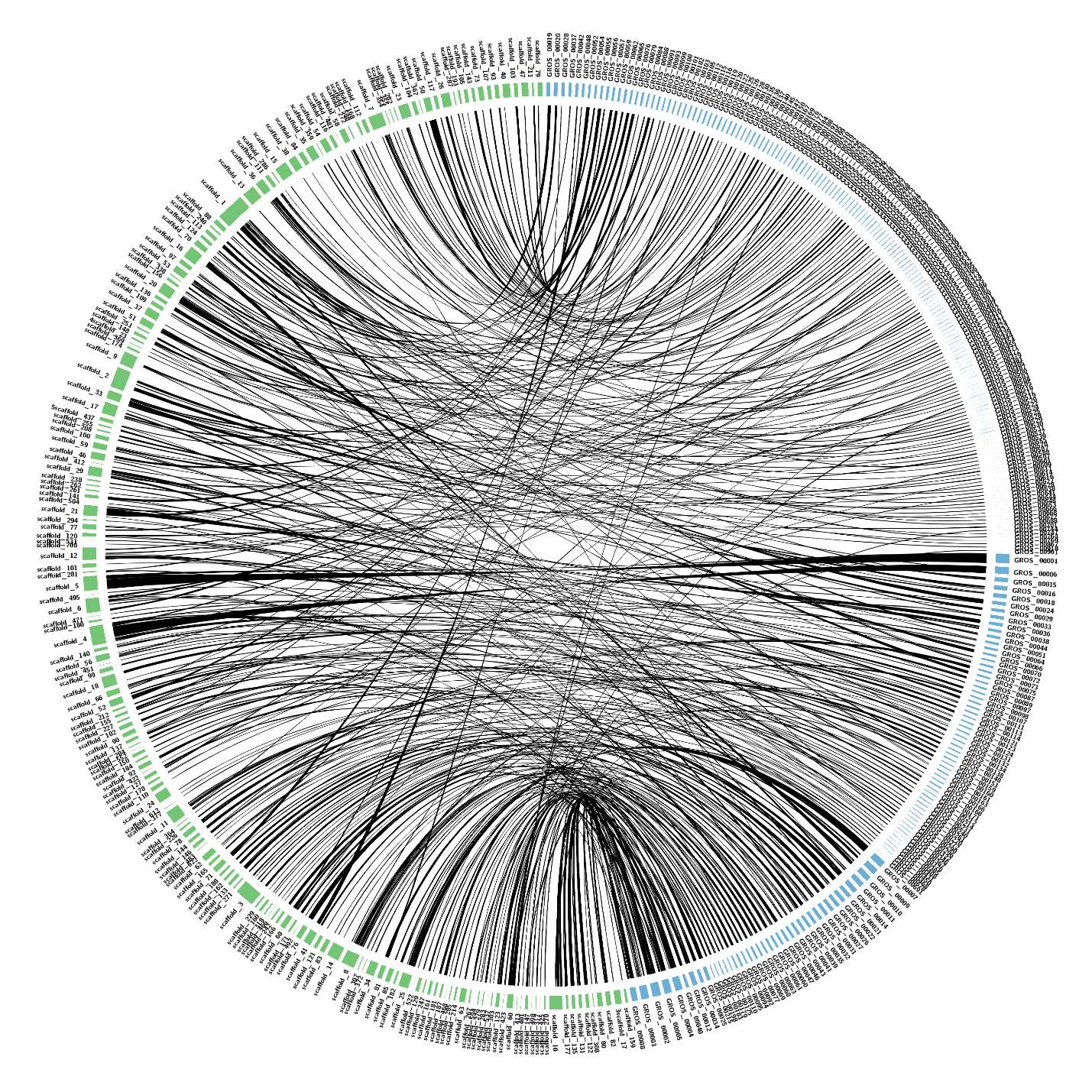


Figure S3


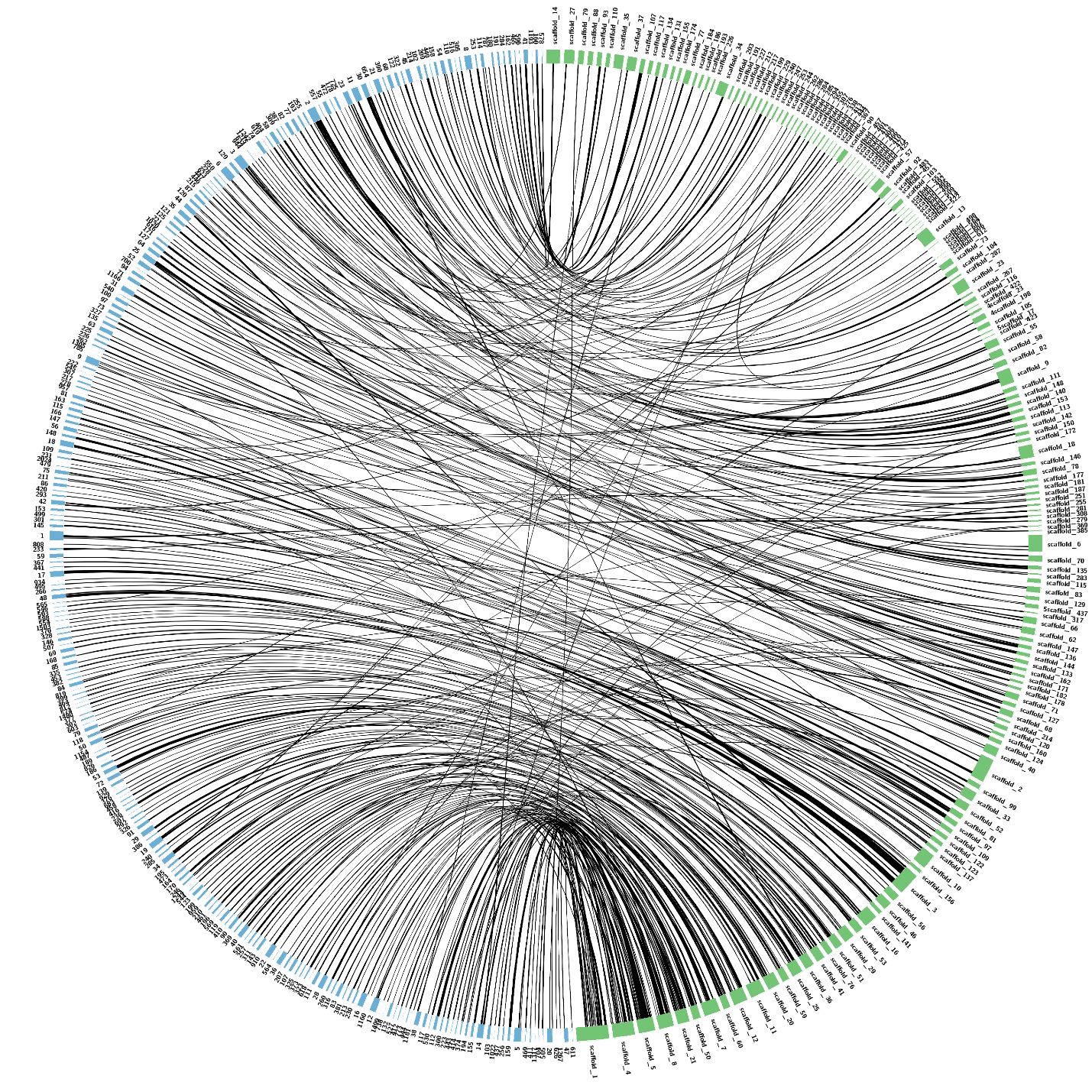


Figure S4


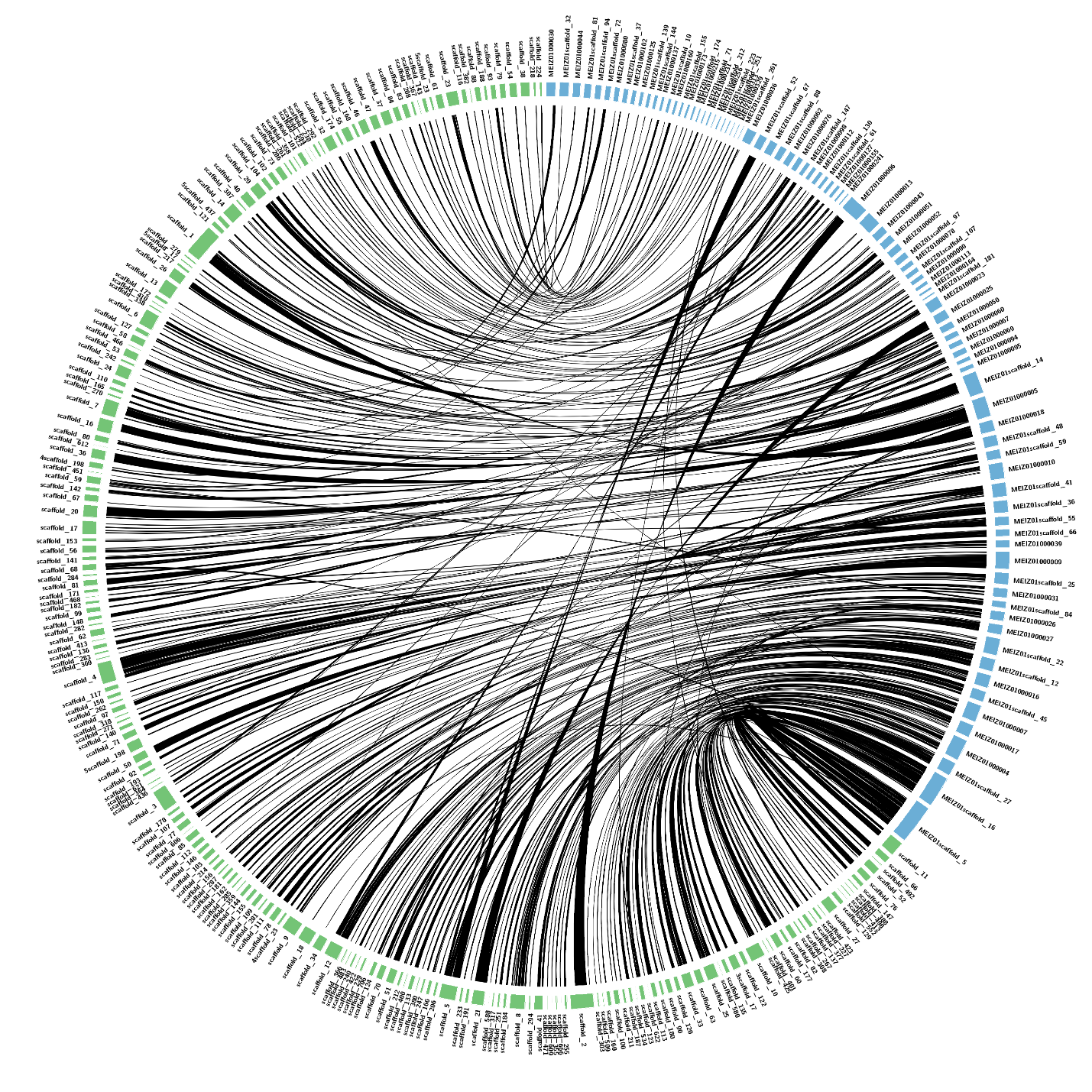


Figure S5


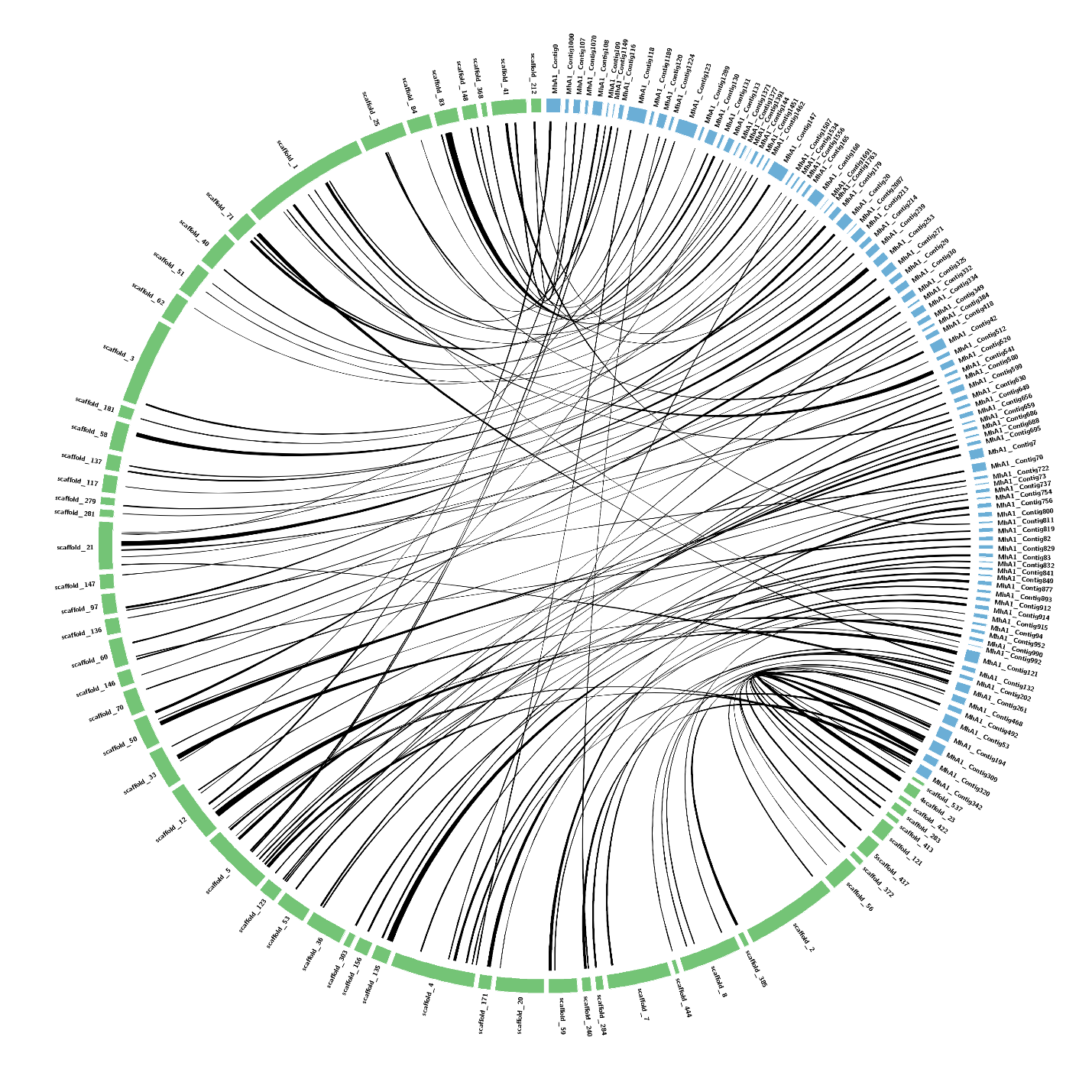


Figure S6


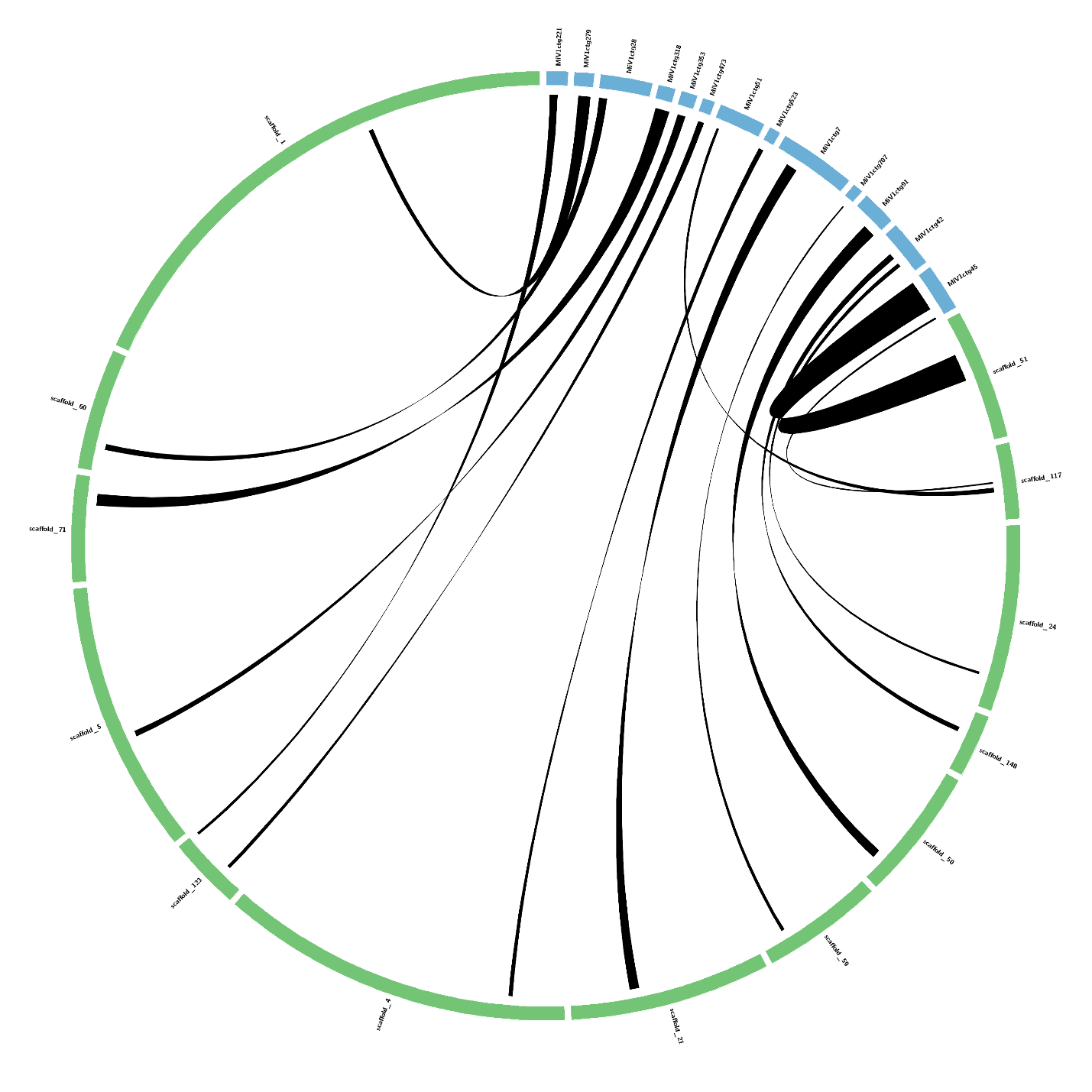


Table S1

|  | **Subreads** | **Preads** | **Ccsreads** | **RNA-seq** | **Transcripts** |
| --- | --- | --- | --- | --- | --- |
| **Total** | 2,382,864 | 326,824 | 89,421 | 301,790,458 | 180,003 |
| **Aligned** | 2,147,202 | 305,042 | 79,359 | 266,166,437 | 155,424 |
| **Percent Alignment** | 90.1% | 93.3% | 88.7% | 88.2% | 86.4% |

Table S2

| **Species** | **Complete** | | **Single copy** | | **Duplicated** | | **Fragmented** | | **Missing** | |
| --- | --- | --- | --- | --- | --- | --- | --- | --- | --- | --- |
| ***B. xylophilus*** | 79.7% | 783 | 77.6% | 762 | 2.1% | 21 | 6.0% | 59 | 14.3% | 140 |
| ***H. glycines*** | 71.9% | 706 | 55.8% | 548 | 16.1% | 158 | 7.9% | 78 | 20.2% | 198 |
| ***G. pallida*** | 50.7% | 498 | 46.6% | 458 | 4.1% | 40 | 9.4% | 92 | 39.9% | 392 |
| ***G. ellingtonae*** | 70.7% | 694 | 67.1% | 659 | 3.6% | 35 | 10.2% | 100 | 19.1% | 188 |
| ***G. rostochiensis*** | 70.7% | 695 | 68.2% | 670 | 2.5% | 25 | 9.4% | 92 | 19.9% | 195 |
| ***M. hapla*** | 59.0% | 580 | 57.0% | 560 | 2.0% | 20 | 9.7% | 95 | 31.3% | 307 |
| ***M. incognita*** | 50.8% | 499 | 34.1% | 335 | 16.7% | 164 | 8.2% | 81 | 41.0% | 402 |

Figure S7


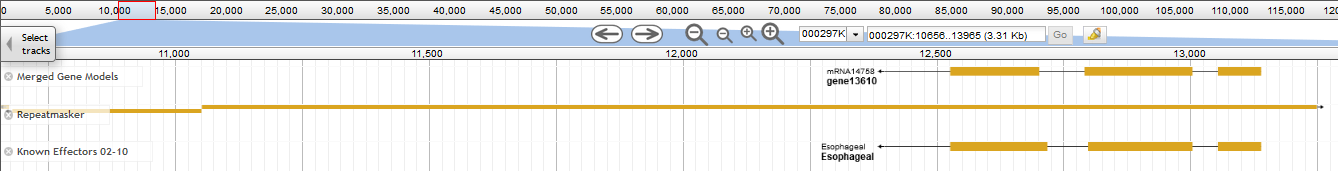


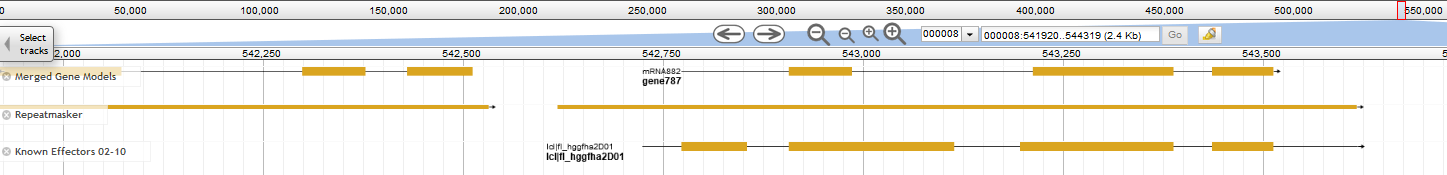


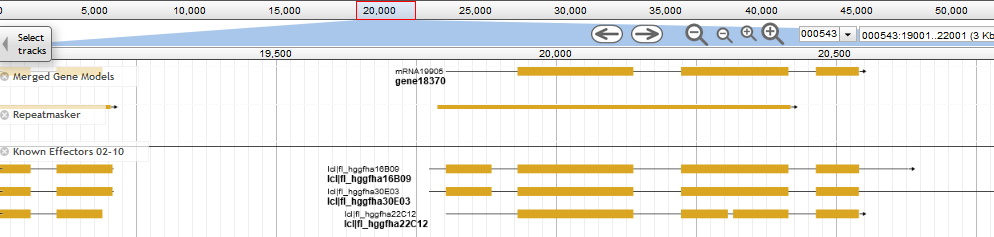


Figure S8


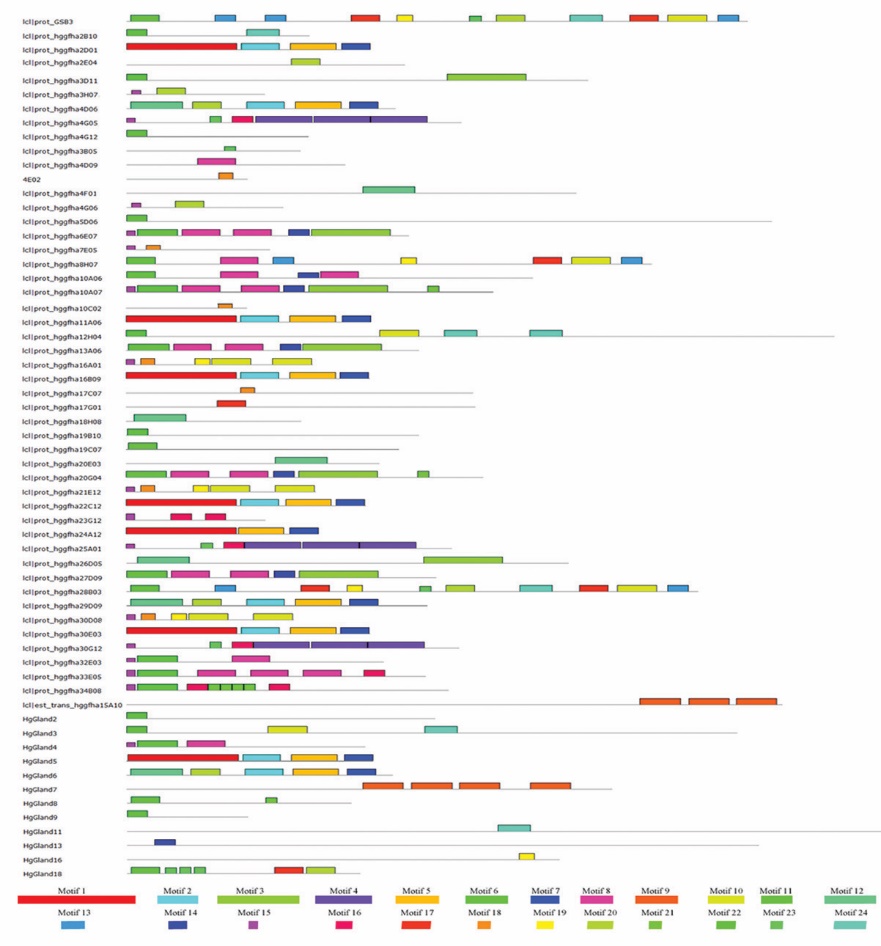


Table S3: *H. glycines* genes previously shown to be acquired by horizontal gene transfer in closely related plant-parasitic nematodes

| **Process** | **Gene family** | **Pfam info** | **Highest AI score** | ***H. glycines* genes** | **Ref.** |
| --- | --- | --- | --- | --- | --- |
| Cell wall degradation | Cellulase | PF00150  Cellulase (glycosyl hydrolase family 5) | 182.1 | G000005019  G000005023  G000005025  G000005226  G000005698  G000006434  G000006440  G000014483  G000015933  G000020774  G000021138  G000021486  G000029557  G000029560  G000029727 | [Smant et al., 1998] |
| Cell wall degradation | Expansin-like proteins | PF03330  Lytic transglycolase | 24.9 | G000008419  G000008425  G000009350  G000021854  G000021855  G000021857  G000021858  G000023597  G000023598  G000027364  G000028153  G000028154 | [Qin et al., 2004] |
| Cell wall degradation | Putative arabinogalactan endo-1,4-beta-galactosidase | PF07745  Glycosyl hydrolase family 53 | 89.2 | G000021138 | [Vanholme et al., 2009] |
| Cell wall degradation | Pectate lyase | PF03211  Pectate lyase | 124.9 | G000000908  G000007021  G000007051  G000010750  G000010754  G000010760  G000016283  G000019886  G000019890  G000021429  G000022761  G000022765  G000022771  G000026629  G000027717  G000027964 | [Kudla et al., 2007] |
| Plant defense manipulation | Chorismate mutase | PF01817  Chorismate mutase type II | 22.4 | G000006353  G000019904  G000020911  G000020912 | [Jones et al., 2003] |
| Plant defense manipulation | Putative isochorismatase | PF00857  Isochorismatase family | 56.9 | G000001356 | [Bauters et al., 2014] |
| Detoxification | Putative cyanate lyase | PF02560  Cyanate lyase C-terminal domain | 37.9 | G000021589 | [Opperman et al., 2008] |
| Nutrient processing | Beta-fructosidase | PF00251  Glycosyl hydrolases family 32 N-terminal domain | 226.7 | G000005632  G000011330  G000013253  G000026283  G000028468  G000028687 | [Cotton et al., 2014; Abad et al., 2008] |
| Vitamin B1 biosynthesis | VB1 thiD | PF08543  Phosphomethylpyrimidine kinase | 49.5 | G000026018  G000026020  G000028057  G000028059 | [Craig et al., 2009] |
| Vitamin B1 biosynthesis | VB1 thiE | PF02581  Thiamine monophosphate synthase | 131.5 | G000026019  G000028058 | [Craig et al., 2009] |
| Vitamin B1 biosynthesis | VB1 thi4 | PF01946¸  Thi4 family | 30.7 | G000009602 | [Craig et al., 2009] |
| Vitamin B1 biosynthesis | VB1 thiM | PF02110  Hydroxyethylthiazole kinase family | 46.0 | G000028057  G000026018 | [Craig et al., 2009] |
| Vitamin B1 biosynthesis | VB1 tenA | PF03070  TENA/THI-4/PQQC family | 87.1 | G000016339 | [Craig et al., 2009] |
| Vitamin B5 biosynthesis | Pantoate ligase | PF02569  Pantoate-beta-alanine ligase | 36.1 | G000006960 | [Craig et al., 2009] |
| Vitamin B6 biosynthesis | Putative pyridoxal 5'-phosphate synthase | PF01680  SOR/SNZ family | 18.6 | G000002105 | [Craig et al., 2008] |
| Vitamin B7 biosynthesis | BioB | PF06968  Biotin synthase | 6.2 | G000006770 | [Paganini et al., 2012] |
|  | Putative polyglutamate synthase | PF09587  Bacterial capsule synthesis protein PGA cap | 28.6 | G000005679 | Veronico et al.,2001 |
|  | Putative glutamine synthase | PF00120  Glutamine synthetase | 6.2 | G000007548  G000023920 | [Scholl et al., 2003; Paganini et al., 2012] |
|  | Putative L-threonine aldolase | PF01212  Beta-eliminating lyase | 83.9 | G000008863  G000008864  G000008869  G000008870 | [Scholl et al., 2003] |
|  | Putative Phosphoribosyl transferase | PF00156  Phosphoribosyl transferase domain | 150.7 | G000004276  G000008373  G000012811  G000012820  G000016458  G000026520 | [Scholl et al., 2003; Paganini et al., 2012] |

Figure S9


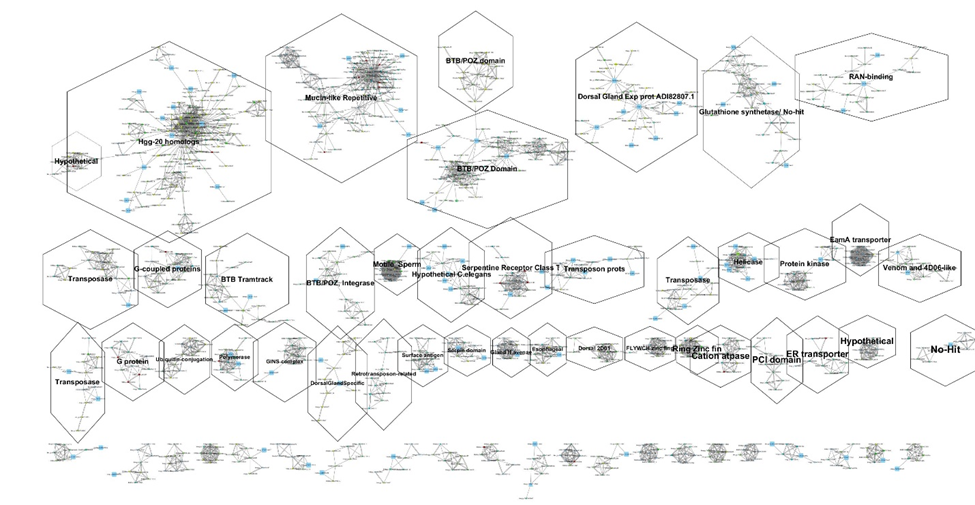


Table S4

|  | **Number of Elements** | **Length Occupied (bp)** | **Percentage of Sequence** |
| --- | --- | --- | --- |
| **SINEs:** | 476 | 54,434 | 0.04% |
| **LINEs:** | 9,035 | 2,266,374 | 1.83% |
| **LINE1** | 1,270 | 163,370 | 0.13% |
| **LINE1** | 193 | 27,800 | 0.02% |
| **L3/CR1** | 3,599 | 1,480,092 | 1.20% |
| **LTR Elements** | 6,584 | 3,621,372 | 2.92% |
| **DNA Elements** | 51,378 | 9,323,978 | 7.53% |
| **hAT-Charlie** | 274 | 30,115 | 0.02% |
| **Unclassified** |  | 23,424,794 | 18.91% |
| **Satellites** | 2,362 | 433,513 | 0.35% |
| **Simple Repeats** | 47,493 | 2,621,602 | 2.12% |
| **Low Complexity** | 14,422 | 1,095,821 | 0.88% |

Figure S10

_
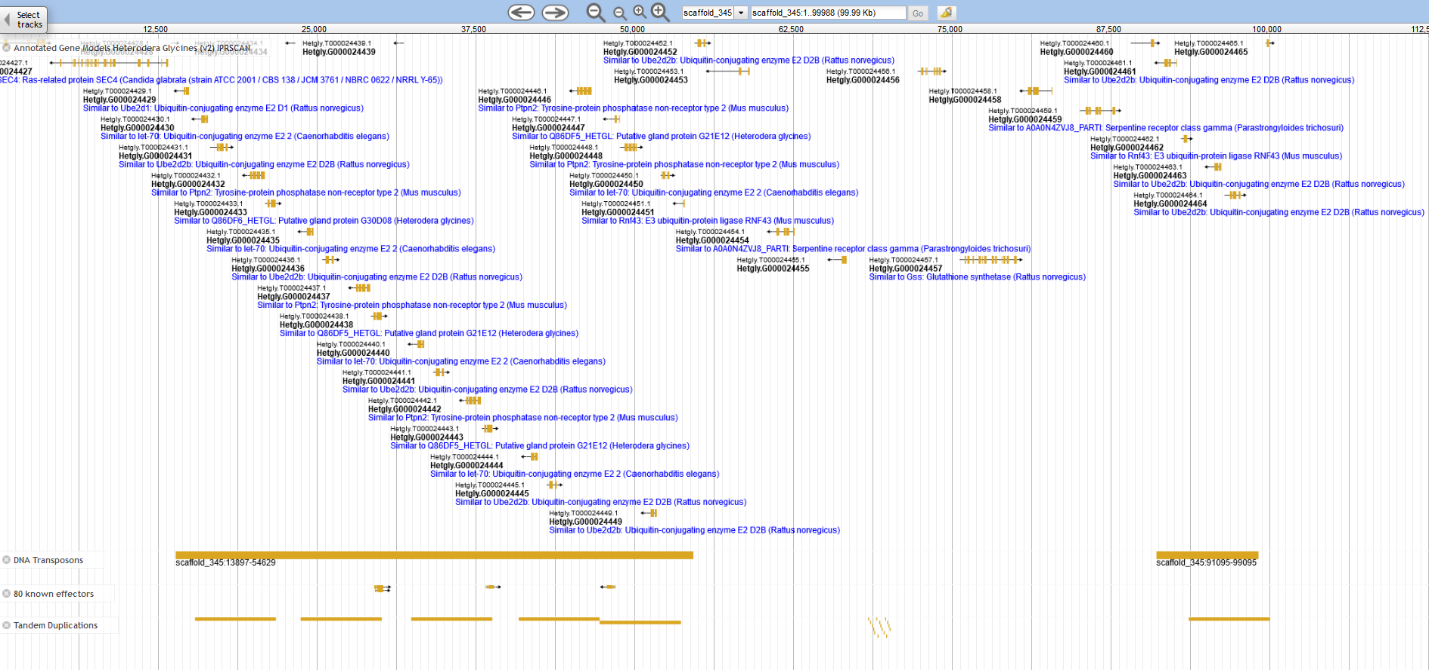
_

A


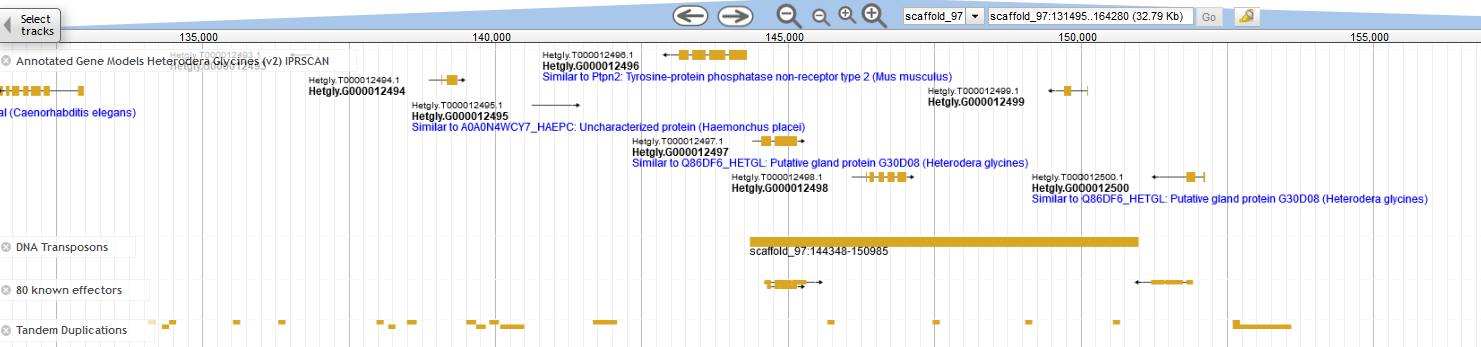


B

Figure S11


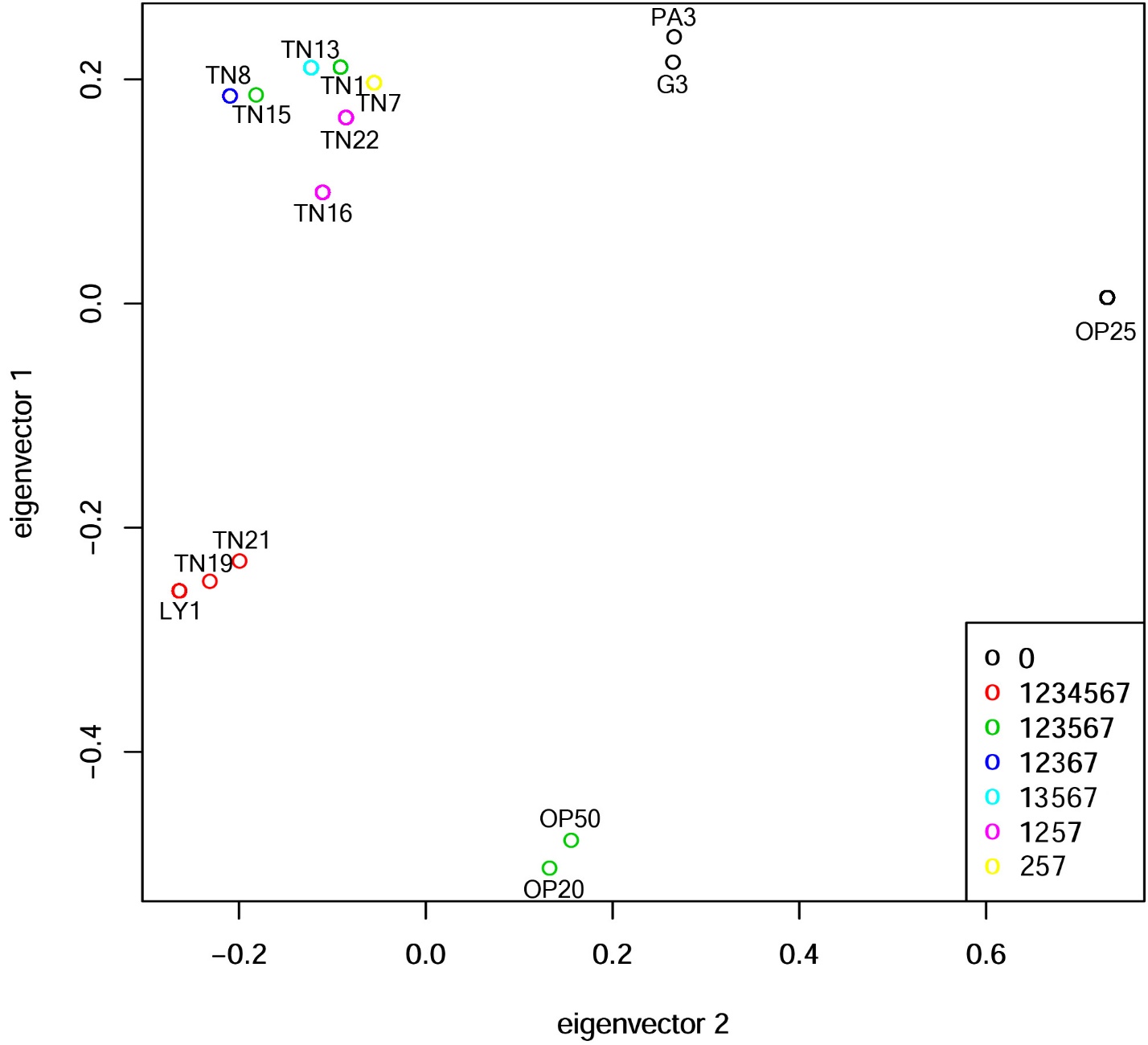


Figure S12


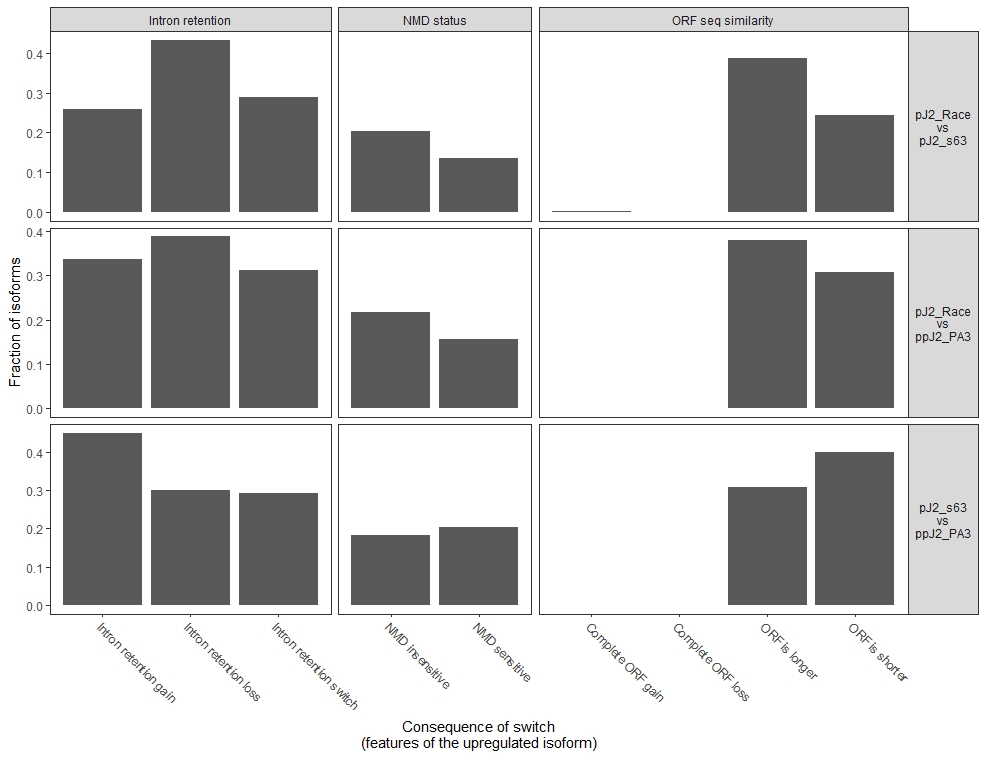


Figure S13


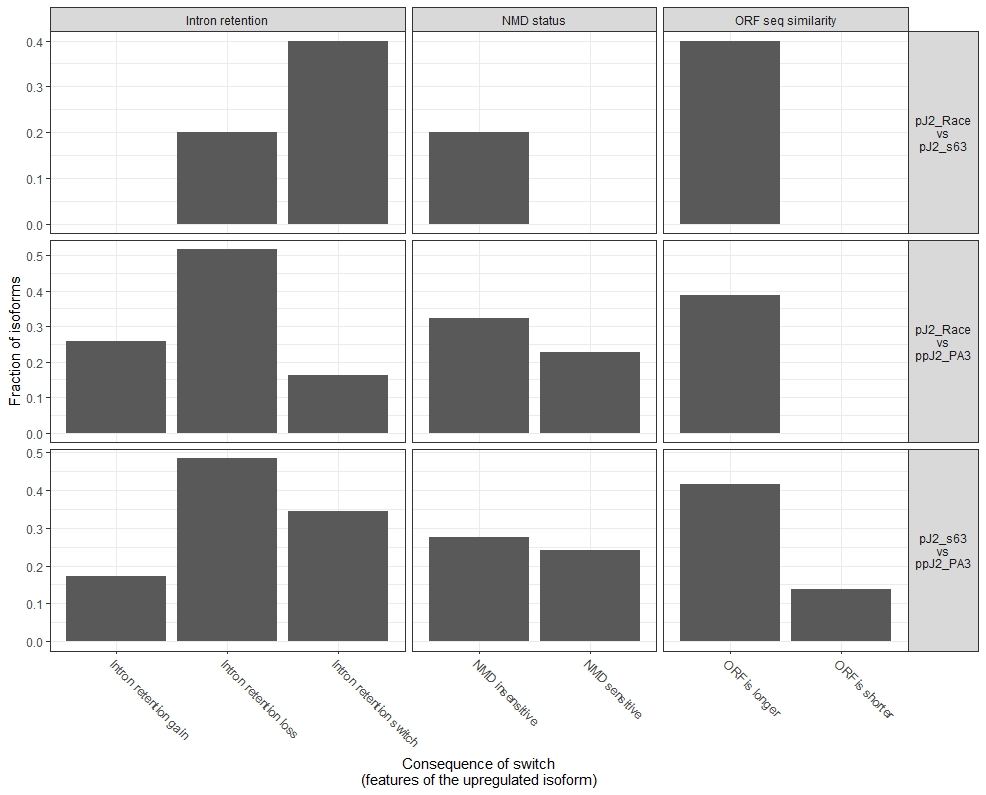

Supplement: Supplementary file 1 — Figure S1. Contamination check with Blobtools. Circles represent scaffolds, while their colors represent different Phyla. All putative contaminating scaffolds are false-positive and have H. glycines origins. The one outlier represents the mitochondrial scaffold, which was misassembled and collapsed to appropriate size. Table S1. Rates of read alignment to the genome for PacBio reads, RNA-seq, and Trinity transcripts. Table S2. Busco genes found in Complete, Single-copy, Duplicated, Fragmented, and Missing categories for assembled genomes in the Tylenchida. Figure S2. Globodera rostchiensis synteny. 439 syntenic regions were identified between G. rostochiensis and H. glycines. Green contigs are H. glycines, while blue contigs are G. rostochiensis. Figure S3. Globodera pallida synteny. 341 syntenic regions were identified between G. pallida and H. glycines. Green contigs are H. glycines, while blue contigs are G. pallida. Figure S4. Globodera ellingtonae synteny. 362 syntenic regions were identified between G. ellingtonae and H. glycines. Green contigs are H. glycines, while blue contigs are G. ellingtonae. Figure S5. Meloidogyne hapla synteny. 112 syntenic regions were identified between M. hapla and H. glycines. Green contigs are H. glycines, while blue contigs are M. hapla. Figure S6. Meloidogyne incognita synteny. 15 syntenic regions were identified between M. incognita and H. glycines. Green contigs are H. glycines, while blue contigs are M. incognita. Figure S7. Repeatmodeler contig alignments overlapping effector alignments in the genome. Three separate examples, with the top track representing final gene models, middle representing Repeatmodeler/Repeatmasker contig alignments, and the lower track representing known effector alignments. Figure S8. Motif analysis of effector sequences. The 80 known effector proteins were subjected to a MEME analysis, and motifs identified in 61 effector proteins implemented with FIMO to find effector candidates in the genome. Ta [file 12864_2019_5485_MOESM1_ESM.docx]
