# Supplementary material for: The genome of the soybean cyst nematode (Heterodera glycines) reveals complex patterns of duplications involved in the evolution of parasitism genes
Source: BMC Genomics. 2019 Feb 7;20:119. doi: 10.1186/s12864-019-5485-8 (PMC6367775; doi:10.1186/s12864-019-5485-8)
Supplement: Supplementary file 7 — Supporting analyses text. (DOCX 22 kb) [file 12864_2019_5485_MOESM7_ESM.docx]

**Splice sites**

The presence of novel GC donor splice sites in *Globodera* species, inspired our efforts to extend the analysis to the *H. glycines* genome (Eves-van den Akker et al. 2016). Because gene prediction with Braker does not assume canonical splicing (Hoff et al. 2015), novel GC donor splicing was found in 8,379 exons across 4.3% of the genes.

**Tandem repeats**

Tandem duplications were not without possible sources of error, as 82/343 were duplicated BUSCO genes. While misassembly could be responsible, or effector duplication may occur at the cost of duplicating genes that are typically maintained at a single copy. 116/431 (27%) predicted effectors are found the 18.6MB (14.6Mb + 4Mb) of consensus tandem duplications, with 98/431 contained in the 14.6 MB reiterated regions. Transposon-mediated duplication is not specific to effectors, as evidenced by 24 and 20 of the 343 duplicated BUSCO genes being contained in DNA and retro-transposons, respectively.

**Repeats**

136 Helitrons were identified using Helitron Scanner, accounting for 1.82% (2.26MB) of the genome. Autonomous helitrons usually encode replication and helicase proteins, thus genic content was assessed revealing 562 genes comprised of 9 helicases, 3 replication-associated proteins, 2 zinc fingers, and 199 proteins with an unknown function.

**Orthologous Genes and Synteny**

Because parasitic nematodes have a reputation for rapidly adapting to unfavorable environments, identifying genes and regions of the genome that are common and unique among different parasitic nematode species may provide some mechanistic insight into the development of resistance. To differentiate conserved genes from those that are extraneous in the *Tylenchida*, orthologous gene families and synteny were calculated between *H. glycines* and *G. pallida*, *G. rostochiensis*, *G. ellingtonae*, *M. hapla*, *M. incognita*, *B. xylophilus*, and *C. elegans* (Figure S2-S6). In total 11,462 genes in the *H. glycines* genome were orthologous to a gene in another species, while 37.8MB of the H. glycines genome is syntenic to another species. The closest related genus, *Globodera,* had the highest number of conserved orthologues and syntenic regions with *H. glycines*, a statistic that declined as phylogenetic distance increased (Figure 1). Further supporting completeness, the number of orthologs identified between *H. glycines* and sister Tylenchida species (~11,500) was similar to the *D. destructor* genome (~11,000) (Zheng et al. 2016), but approximately 3000 more than found in *Globodera* species(Eves-van den Akker et al. 2016).

**HGT**

A small number of genes previously hypothesized to be acquired from HGT, were found but yielded very low AI scores (Supplemental Data 2). This was the case for chitinases (PF00704) and two protein families possibly involved in feeding site induction and coding for acetyltransferase: Maltose acetyltransferase (PF12464) and Bacterial transferase hexapeptide (PF00132) that were reported by Scholl et al. 2003 and Clifton et al. 2003. These genes were all identified in the *H. glycines* genome, but their AI scores did not support an HGT origin (Supplemental Data 2). For example, at least eleven occurrences of Glycosyl hydrolases family 18 (chitinase) were found with AI ranging from -16.4 (Hetgly.000018874) to -157.7 (Hetgly.000019682), strongly suggesting a Metazoan origin. Similarly, nine different genes were found for the two acetyltransferases without HGT evidence (AI from -65.2 to 0). However, several other acetyltransferase with strong AI (up to 43.1) were found, for example three esophageal gland-localized secretory protein 1 (Hetgly.000020742, Hetgly.000016876, and Hetgly.000020731).

Of particular interest were those with strong BLAST hits in bacteria or fungi and no hit on Metazoa (indicated in blue in Supplemental Data 3). Among these were two genes coding for an Inosine-uridine preferring nucleoside hydrolase (Hetgly.G000014320, Hetgly.G000014322; AI up to 122.16). This enzyme is essential for parasitism in many plant pathogenic bacteria and trypanosomes [Gopaul et al., 1996]. The protein RxLR, a candidate effector in an oomycete [Morgan & Kamoun, 2007], was also identified in H. glycines (Hetgly.G000019006; AI=37.92). Besides being necessary for successful infection, RxLR is also an Avr gene in some species, including the soybean pathogen Phytophthora sojae [Shan et al., 2004]. The G. pallida effector protein Gp-FAR-1 was also identified as a HGT in the H. glycines genome (Hetgly.G000027954 and Hetgly.G000029399; AI=42.92). This protein is thought to be involved in plant defense evasion because it binds to lipid precursors of plant defense compounds and the jasmonic acid signaling pathway [Prior et al., 2001].

Two genes from the dienelactone hydrolase family were identified (Hetgly.G000019465 and Hetgly.G000009919; AI up to 156.31) and are also involved in virulence in the cereal pathogen *Fusarium pseudograminearum*, but absent in avirulent species [Gardiner et al., 2012]. The closet hits to the H. glycines proteins came from Rhizobium species, an HGT donor suspect to PPN that inhabits the same ecological niche. Genes similar to the Rhizobia NodL genes encoding an N-acetyltransferase involved in the biosynthetic pathway of Nod factors were already highlighted in PPN [Scholl et al., 2003]. In this study, we identified several SAM-dependent methyltransferases (e.g. Hetgly.G000000773; AI= 139.82) similar to NodS [Geelen et al., 1995]. Interestingly, the SCN resistance locus Rhg4 also encodes a methyltransferase and the transgenic overexpression in soybean of GmSAMT1, also a SAM-dependent methyltransferase, resulted in resistance to H. glycines [Lin et al., 2016]. Other genes with best hits from Rhizobia and lacking a hit to Metazoan sequences, include two glycosyl transferase group 1 proteins (Hetgly.G000014320, Hetgly.G000014322; AI up to 122.16), a DNA ligase D.

**Alternative splicing**

The most abundant alternative splicing events were intron retention (30%) and non-mediated decay (15%), with 70% of alternative splicing events changing open reading frame length (Figure S12 & S13). In comparison, previous work utilizing a de novo transcriptome assembly approach discovered 71,093 genes with 147,910 (2.08 average) transcripts in *H. glycines* (Gardner et al. 2018) thus demonstrating the importance of using an assembled genome as a part of the transcriptomics pipeline (Lee and Rio 2015).

To gain further insights into the prevalence of alternative splicing within known effector proteins, the 80 previously identified effector proteins were associated with 371 transcripts. This differs from previous work where 395 transcripts were associated with the 80 effectors in a de-novo transcriptome approach (Gardner et al. 2018). The main types of alternatively spliced variants for the effector genes included 73 (19.7%) intron retention, 26 (7.0%) alternative 5’ donor site, 25 (6.7%) alternative 3’ acceptor site, 43 (11.6%) alternative transcription start site, 47 (12.7%) alternative transcription termination site, 4 (1.1%) single exon skipping, and 30 (8.1%) multiple exon skipping.

To explore effects that alternative splicing may have on effector protein function, a functional domain analysis was conducted using the Pfam domain annotation tool (Finn et al. 2015). Of the 69/80 single copy effectors genes, only nine (7.7%) with 51 corresponding isoforms had functional protein domains. Within these, twelve protein functional domains were identified (~0.24 domains/isoform) with at least one AS event altering domain architecture resulting in at least one added, modified, or deleted functional domain.

**PCA analysis**

To better understand genomic associations among differing HG types, a PCA plot was constructed for the 15 sequenced populations of SCN. Interestingly supervirulent populations created unique clusters, while all other types of virulence were less associated (Figure S8, Table S6). Pa3 and G3 populations (black) are both avirulent and started from similar populations of “Race 3” nematodes. All three super-virulent populations, LY1, TN19, TN21 (red) clustered together, even though TN21 was obtained from a different field isolate. The OP50 and OP20 populations (green) and OP25 (black) started from the same NC field isolate, but were subject to different selective pressures. OP50 was selected on PI90763 resistance, while OP20 was selected on PI88788 resistance, both resulting in a HG type of 1.2.3.5.6.7. However, when the same NC population was not exposed to selective pressure OP25(yellow), an Hg type 1.5.7 resulted. In the largest cluster five of seven populations are related by heritage, as TN1 was the progenitor population of TN7, TN8, TN13 and TN15. The commonality among all seven populations lies with their common selection across a variation of SCN resistant cultivars, resulting in HG types ranging from 1.3.5.7 to 1.2.3.5.6.7.

Eves-van den Akker S, Laetsch DR, Thorpe P, Lilley CJ, Danchin EG, Da Rocha M, Rancurel C, Holroyd NE, Cotton JA, Szitenberg A (2016) The genome of the yellow potato cyst nematode, Globodera rostochiensis, reveals insights into the basis of parasitism and virulence. Genome biology 17:124

Finn RD, Coggill P, Eberhardt RY, Eddy SR, Mistry J, Mitchell AL, Potter SC, Punta M, Qureshi M, Sangrador-Vegas A (2015) The Pfam protein families database: towards a more sustainable future. Nucleic acids research 44:D279-D285

Gardner M, Dhroso A, Johnson N, Davis EL, Baum TJ, Korkin D, Mitchum MG (2018) Novel global effector mining from the transcriptome of early life stages of the soybean cyst nematode Heterodera glycines. Scientific reports 8:2505

Hoff KJ, Lange S, Lomsadze A, Borodovsky M, Stanke M (2015) BRAKER1: unsupervised RNA-Seq-based genome annotation with GeneMark-ET and AUGUSTUS. Bioinformatics:btv661

Lee Y, Rio DC (2015) Mechanisms and regulation of alternative pre-mRNA splicing. Annual review of biochemistry 84:291-323

Zheng J, Peng D, Chen L, Liu H, Chen F, Xu M, Ju S, Ruan L, Sun M (2016) The Ditylenchus destructor genome provides new insights into the evolution of plant parasitic nematodes. Proc R Soc B. The Royal Society, p 20160942
